# Three-dimensional organ segmentation and structural phenotyping of salt-stressed coriander seedlings using the optimized point transformer-based model PTV-SegCo

**DOI:** 10.3389/fpls.2026.1883203

**Published:** 2026-08-31

**Authors:** Shihan Wang, Yujuan Xu, Erpeng Cheng, Jun’ao Cheng, Siying Liu, Yuanyu Xia, Ziyi Zhang, Xiuqing Fu

**Affiliations:** College of Engineering, Nanjing Agricultural University, Nanjing, China

**Keywords:** coriander seedlings, salt stress, semantic segmentation, structural phenotyping, three-dimensional point cloud

## Abstract

Salt stress can markedly alter seedling architecture, creating a need for non-destructive three-dimensional (3D) phenotyping methods capable of resolving fine plant structures. However, organ-level segmentation of plant point clouds remains challenging because of leaf overlap, slender stems, ambiguous stem–leaf boundaries, and severe class imbalance. In this study, we developed PTV-SegCo, a task-adapted Point Transformer model for organ segmentation and structural phenotyping of coriander seedlings under salt stress. PTV-SegCo integrates efficient channel attention, gated shallow–deep feature fusion, and a combined cross-entropy–Dice loss to improve representation of fine and minority organ structures. The dataset comprised 60 manually annotated 3D point-cloud samples from 12 cultivation trays repeatedly observed over five acquisition dates under six NaCl concentrations (0, 50, 100, 150, 200, and 250 mmol L^−1^). Because the earliest acquisition represented a particularly challenging developmental stage, these 12 samples were used as a fixed early-stage model-selection set, while samples from the remaining four dates were organized into four date-blocked training–validation configurations. Under this internal model-development protocol, PTV-SegCo achieved mean mAcc and mIoU values of 93.05% and 89.05%, respectively, and showed numerically higher performance than its direct backbone PTV-Seg50. These values should be interpreted as internal comparative results rather than as an unbiased estimate of generalization to unseen cultivation trays, and the present results should not be interpreted as establishing the broad competitiveness of PTV-SegCo against other point-based, convolution-based, graph-based, transformer-based, or plant-specific segmentation architectures. After semantic segmentation, reconstructed scenes were metrically calibrated using the known cultivation-tray dimensions, followed by individual-plant separation and quality control. Four reconstruction-derived structural descriptors—plant height, projected area, voxel occupancy volume, and leaf point ratio—were extracted to characterize temporal structural variation under different NaCl treatments. For treatment-level inference, individual-plant measurements were aggregated within each cultivation tray at each acquisition time, with the cultivation tray treated as the independent experimental unit. Independent manual validation showed close agreement for plant height and projected area, with R^2^ values of 0.9969 and 0.986, respectively. Overall, the proposed workflow provides a feasible approach for organ-level segmentation and automated 3D structural analysis of small coriander seedlings under salt stress. The extracted descriptors primarily represent reconstruction-derived spatial characteristics and should not be interpreted as direct indicators of physiological status; voxel occupancy volume and leaf point ratio remain without direct external validation.

## 1 Introduction

Coriander is an important culinary vegetable and economic crop with high nutritional value and various bioactive compounds, and it has a wide consumption demand worldwide (Wei et al., 2019). However, soil salinization has become one of the major environmental factors restricting sustainable agricultural development. The global area affected by soil salinity continues to expand, causing severe impacts on crop growth, development, and yield formation (FAO, 2021; Shokri et al., 2024). Salt stress mainly affects normal plant growth through osmotic stress, ion toxicity, and oxidative damage, resulting in phenotypic responses such as restricted leaf expansion, altered plant architecture, and reduced biomass accumulation (Waheed et al., 2024; Zhou et al., 2024).Previous studies on coriander have demonstrated that increased salinity levels can inhibit plant height, leaf number, biomass accumulation, or yield formation under specific experimental conditions, accompanied by variations in certain physiological and biochemical parameters (Alghabban et al., 2023; Brengi et al., 2024). However, the experimental conditions and measured traits in these studies are not completely consistent with those of the present study. In this work, the structural phenotypic changes of coriander seedlings were characterized solely based on three-dimensional (3D) point cloud data, and no direct inference was made regarding specific physiological mechanisms. For coriander seedlings at the early growth stage, morphological changes during development can reflect structural responses under salt stress conditions. Therefore, continuous acquisition of plant growth information is valuable for analyzing structural variations under different salinity treatments. Currently, plant phenotyping still relies largely on manual measurements, which generally suffer from low efficiency, subjective bias, and difficulties in achieving continuous and non-destructive monitoring (Xiao et al., 2022; Li et al., 2026). Although 3D plant segmentation techniques have developed rapidly in recent years, their applicability for continuous and non-destructive structural analysis of salt-stressed coriander seedlings has not been sufficiently validated.

In recent years, advances in computer vision technologies have promoted the development of high-throughput plant phenotyping. Two-dimensional (2D) image-based approaches, including RGB imaging, multispectral imaging, and thermal infrared imaging, have been widely applied for evaluating plant growth status and stress responses (Fahlgren et al., 2015; Gill et al., 2022). Meanwhile, deep learning methods such as U-Net and Mask R-CNN have further improved plant image segmentation and phenotypic trait extraction capabilities (Praveen Kumar and Domnic, 2019; Vayssade et al., 2022). However, for small vegetable crops such as coriander seedlings, which typically possess fragile leaves, complex fine-scale structures, and frequent leaf overlap, 2D projections inevitably lose depth information and spatial topological relationships. Leaf occlusion, organ overlapping, and unclear boundaries between stems and leaves may further reduce the accuracy of individual plant separation and organ-level phenotypic trait extraction (Gill et al., 2022; Vayssade et al., 2022). In contrast, 3D point clouds can directly represent plant spatial structures and topological relationships, thereby reducing information loss caused by 2D projection and providing a promising approach for analyzing complex plant architectures (Harandi et al., 2023; Song et al., 2025). Recent reviews and comparative studies on organ-level plant point cloud segmentation have indicated that segmentation performance is influenced by multiple factors, including point cloud acquisition environments, sensor types, plant species, and model selection. The performance of a given model is not necessarily consistent across different datasets (Xie et al., 2024). Therefore, the availability of 3D data alone does not guarantee stable organ recognition performance, and task-specific adaptation according to crop architecture and acquisition conditions remains necessary.

Three-dimensional point cloud analysis techniques have developed rapidly in recent years and have gradually been applied to plant organ recognition and structural phenotypic trait extraction. PointNet and PointNet++ demonstrated that deep learning models can directly learn spatial features from unordered point sets, providing fundamental frameworks for semantic segmentation of plant point clouds (Charles et al., 2017; Qi et al., 2017). Subsequently, researchers improved the capability of point cloud models to represent plant spatial structures and complex boundaries by incorporating strategies such as local geometric feature learning, multi-scale feature fusion, and attention mechanisms. These developments have facilitated organ-level point cloud segmentation studies for various plant species. For example, previous studies have proposed task-oriented improvements targeting seedling structure analysis, differences in organ scales, and complex plant morphologies (Zhang et al., 2024; Liu et al., 2025; Qiao et al., 2025). However, existing studies have demonstrated that plant point cloud segmentation is highly task-dependent, with performance affected by plant morphology, organ categories, data acquisition methods, and class distribution characteristics. For small dicotyledonous seedlings, current segmentation methods still face considerable challenges due to dense leaf arrangements, substantial differences in organ scales, slender stems, and unclear boundaries between stems and leaves. In particular, structures with relatively few points, such as stems, petioles, and nodes, are highly susceptible to class imbalance, resulting in reduced segmentation performance for minority categories (Boogaard et al., 2022). Meanwhile, previous research on pumpkin seedlings has also shown that slender stem structures are easily affected by soil and leaf occlusion, and ambiguous boundaries further increase recognition difficulty (Deng et al., 2024). Therefore, for complex small dicotyledonous plants such as coriander seedlings, developing task-adapted point cloud segmentation strategies according to their specific structural characteristics is essential for improving the recognition stability of fine organs and regions with ambiguous boundaries.

However, previous studies have mainly focused on general plant organ segmentation or 3D reconstruction tasks, whereas systematic research on 3D point cloud-based phenotypic analysis of coriander seedlings under salt stress remains limited. Therefore, the main contribution of this study is to establish a task-oriented 3D point cloud phenotyping framework for salt-stressed coriander seedlings. Rather than proposing an entirely new point cloud backbone architecture, this framework integrates dataset construction, model adaptation for specific plant structures, and automated extraction of structural phenotypic traits. Compared with general-purpose point cloud segmentation models, this study primarily focuses on improving structural adaptability for organ-level segmentation of small plant seedlings. Based on this framework, a complete workflow from multi-view 3D reconstruction, semantic segmentation, individual plant separation, to automated structural phenotypic trait extraction was established to characterize structural changes in coriander seedlings under different NaCl treatments. Because PTV-SegCo was developed as a task-oriented adaptation of PTV-Seg50, PTV-Seg50 was selected as the direct controlled baseline. This comparison was intended to isolate the effects of the introduced ECA module, gated feature fusion strategy, and joint Cross-Entropy–Dice loss under the same backbone framework and experimental settings. It was not designed as a comprehensive benchmark across different point-cloud segmentation model families. Therefore, the present comparison cannot establish the broad competitiveness of PTV-SegCo against classical point-based, graph- or convolution-based, recent transformer-based, or plant-specific segmentation methods. Such systematic benchmarking remains necessary in future studies using unified cultivation-tray-grouped partitions and identical training and evaluation settings. Specifically, based on the PTV-Seg50 framework, this study introduced an efficient channel attention mechanism, a gated shallow–deep feature fusion module, and a combined cross-entropy and Dice loss function to address the challenges associated with coriander seedling point clouds, including low proportions of fine stem points, severe leaf overlap, and unclear boundaries between stems and leaves. These improvements were designed to enhance the recognition capability of key organs and minority structural categories. Furthermore, based on the semantic segmentation results, this study extracted several 3D structural phenotypic traits, including plant height, projected area, voxel occupancy volume, and leaf point ratio, to characterize early structural changes in coriander seedlings under different NaCl concentrations. The stem region serves as an important structure connecting the root system and leaves, and improving its segmentation accuracy can help reduce errors in plant height estimation and overall spatial structure analysis, thereby enhancing the reliability of growth response analysis under different salt treatment conditions. It should be emphasized that these parameters mainly represent structural characteristics derived from 3D reconstruction and segmentation results. In the present study, plant height and projected area were additionally evaluated against independent manual reference measurements to assess their measurement accuracy, whereas voxel occupancy volume and leaf point ratio remain reconstruction-derived descriptors without direct external validation. Accordingly, the subsequent analyses are restricted to the structural phenotypic level and are not used to infer specific physiological mechanisms. This study aims to provide a feasible task-adaptation strategy and workflow for 3D point cloud analysis of complex structures in small vegetable seedlings. Although PTV-SegCo produced numerically higher segmentation metrics than PTV-Seg50 under the internal model-development protocol used in this study, the present work mainly focused on task-oriented adaptation of the Point Transformer framework for coriander seedling point cloud segmentation. Future research should further evaluate the proposed approach through systematic comparisons with more general point cloud segmentation architectures and plant-specific segmentation methods using larger annotated datasets.

The main contributions of this study are summarized as follows:

1. A dataset containing 60 3D point cloud samples of coriander seedlings was constructed, covering six NaCl concentrations (0, 50, 100, 150, 200, and 250 mmol L^−1^) and continuous acquisition processes. This dataset provides experimental support for 3D structural analysis of salt-stressed coriander seedlings under the conditions investigated in this study.
2. Considering the characteristics of coriander seedling point clouds, including low proportions of fine stem points, severe leaf overlap, and ambiguous stem–leaf boundaries, a task-oriented model adaptation strategy was developed based on PTV-Seg50. The effect of this adaptation on organ-level segmentation performance was examined through internal ablation and comparative analyses using the constructed dataset.
3. An automated workflow for extracting 3D structural parameters based on semantic segmentation results was established to quantitatively describe spatial structural changes in coriander seedlings under salt treatments. The extracted traits were restricted to structural parameters derived from point cloud reconstruction results.
4. Structural trait variation trends of coriander seedlings under different NaCl concentrations were analyzed using continuous observation data. The conclusions were limited to the dataset, acquisition conditions, and structural parameters investigated in this study, providing a reference for future external validation and expanded research.

## 2 Materials and methods

### 2.1 Experimental equipment and experimental design

To obtain three-dimensional (3D) phenotypic information of coriander seedlings under salt stress conditions, a controlled indoor cultivation experiment was conducted in this study. Seeds of coriander were selected as the experimental materials, and six NaCl concentration treatments were established, including 0, 50, 100, 150, 200, and 250 mmol L^−1^. Two independent cultivation trays were prepared for each NaCl concentration treatment. Twenty-five seeds were sown in each cultivation tray and arranged in a 5 × 5 grid pattern, resulting in a total of 12 cultivation trays. During the experiment, plants from all treatment groups were cultivated under identical growth conditions. The cultivation tray was considered the experimental unit for salt stress treatment analysis to ensure the rationality of the experimental design and subsequent statistical analyses. The main environmental parameters and experimental conditions during cultivation, including the cultivation environment, containers, planting number, NaCl concentrations, and environmental control parameters, are provided in **Table 1**.

**Table 1:** Experimental cultivation conditions of coriander seedlings.

Parameter	Setting
Plant species	Coriandrum sativum L.
Cultivation environment	Indoor controlled growth chamber
Cultivation container	Acrylic tray (25 cm × 25 cm × 5 cm)
Number of seeds per tray	25
Sowing pattern	5 × 5 grid
NaCl treatments	0, 50, 100, 150, 200, 250 mmol/L
Number of trays per treatment	2
Cultivation substrate	horticultural nutrient soil
Temperature	23 ± 1 °C
Relative humidity	60 ± 5%
Photoperiod	16 h light/8 h dark
Light intensity	200 μmol m^−2^ s^−1^
Salt application time	From the first day after sowing, twice daily irrigation with corresponding NaCl solutions

The experimental system mainly consisted of a controlled cultivation environment and a multi-view image acquisition system. The controlled cultivation environment was used to provide stable temperature, relative humidity, and light conditions, thereby reducing the influence of environmental variations on plant growth. To obtain complete spatial structural information of coriander seedlings, a multi-view imaging system was established, consisting of an industrial camera (MV-CS200-10GC, 5472 × 3648 pixels), an electric rotary platform, LED supplementary lighting sources, and a synchronized control module. The detailed system configuration is presented in **Table 2**.

**Table 2:** Multi-view imaging system configuration.

Component	Specification	Purpose
Camera	MV-CS200-10GC, 5472×3648 px	Image acquisition
Rotation stage	Electric rotary stage	Multi-angle imaging
Lighting	LED light source	Stable illumination
Control unit	Custom control system	Synchronization
Acquisition views	Front view and axonometric view	3D reconstruction

**Figure 1:**
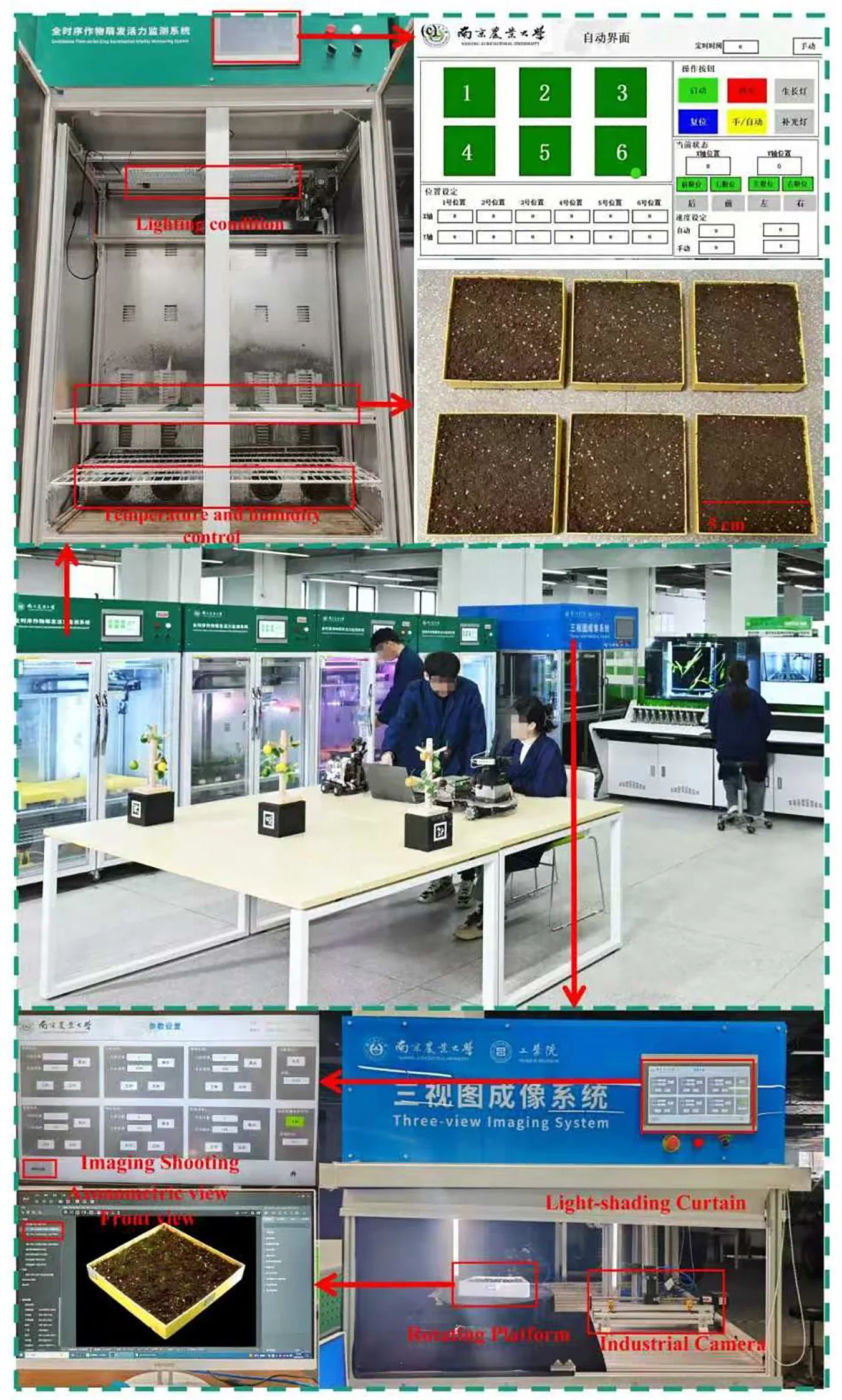
Experimental procedure.

It should be noted that although the imaging platform used in this study had multi-view acquisition capability, only two views were collected during the actual image acquisition process, including the front view and the axonometric view. By integrating information from different viewpoints, the coverage of overlapping leaf regions and fine stem structures was improved, thereby reducing the influence of occlusion on the completeness of 3D reconstruction. The experimental workflow is shown in **Figure 1**, which mainly included seed pre-treatment, sowing and cultivation, salt stress treatment, multi-view image acquisition, 3D point cloud generation, and manual annotation. During the experiment, image data of coriander seedlings under different salt concentrations were continuously collected, followed by 3D reconstruction and manual annotation to construct a 3D point cloud dataset for subsequent semantic segmentation tasks.

### 2.2 Image acquisition and 3D point cloud reconstruction

To obtain the three-dimensional (3D) spatial structural information of coriander seedlings under salt stress conditions, a multi-view image acquisition method was adopted to collect plant image data, and 3D point clouds were reconstructed based on the image sequences. During the experiment, video sequences from the front view and axonometric view were collected for each cultivation tray. The video acquisition frame rate was set to 8.8 fps, and the duration of each video was approximately 107 s. According to the angular variation of the rotary platform, one image frame was extracted from the video every 6° of rotation; therefore, 60 multi-view images were obtained during each complete rotation cycle. The collected multi-view images were reconstructed into 3D point clouds using COLMAP software. First, the Structure-from-Motion (SfM) module was used to perform feature matching among images from different viewpoints and estimate camera poses to establish a sparse 3D structure (Schönberger and Frahm, 2016). Subsequently, the Multi-View Stereo (MVS) module was applied for dense reconstruction to generate 3D point cloud data containing spatial coordinate information (Schönberger et al., 2016). In this study, the default SfM and MVS workflows of COLMAP were adopted for point cloud generation without additional adjustment of internal reconstruction parameters. During the reconstruction process, camera intrinsic and extrinsic parameters were automatically estimated by COLMAP based on the input images and were used for camera pose estimation and dense point cloud generation.

The reconstructed point clouds retained the original coordinate relationships generated by COLMAP and were not calibrated using an external scale reference at the reconstruction stage. Therefore, the coordinates used for dataset construction, semantic annotation, model training, and semantic segmentation represented relative spatial coordinates rather than absolute physical measurement units. This relative-coordinate representation was sufficient for the semantic segmentation task because the model was designed to learn spatial and appearance features for organ classification rather than to directly estimate physical dimensions. Importantly, the reconstruction-scale point clouds used for semantic segmentation and the point clouds used for quantitative structural phenotyping were treated separately in the subsequent workflow. The original reconstruction coordinate system was retained throughout model development and evaluation, whereas an additional metric calibration procedure was applied only after semantic segmentation and before quantitative phenotypic trait extraction. The metric calibration procedure is described separately in Section 2.4.1.

To ensure the consistency and reliability of point cloud annotation, a unified annotation protocol was established in this study. The pot category was mainly identified based on its distinct boundary structures and spatial position. The stem category was determined according to its slender cylindrical structural characteristics and connection relationships with leaves. The leaf category was identified based on its planar structural characteristics and spatial distribution. For regions with unclear boundaries, such as stem–leaf junction areas, manual decisions were made by considering multi-view observations of the 3D point clouds, spatial continuity, and the overall plant morphology. All point cloud samples were initially annotated by the same researcher, followed by independent inspection and correction by another researcher with experience in 3D point cloud processing. For regions with uncertain class boundaries or potential mislabeling, the annotations were reviewed and adjusted by integrating the original image information with the 3D structural information. Finally, a manually annotated 3D point cloud dataset was obtained for model training and evaluation. To evaluate the influence of manual preprocessing on the number of point cloud data points, the changes in point numbers before and after processing were statistically analyzed for all 60 samples. The results showed that the average number of points before preprocessing was approximately 1.148 × 10^6^ points/sample, while the average number after preprocessing was approximately 1.143 × 10^6^ points/sample, corresponding to an average reduction ratio of approximately 0.43%. These results indicate that background removal and noise cleaning mainly affected regions outside the cultivation tray and a small number of discrete noise points, while having limited influence on the structural information of coriander seedlings.

Before being used as model inputs, voxel sampling was applied to reduce point cloud redundancy and improve training efficiency. Because metric calibration was not applied during model development, the voxel size used for semantic segmentation preprocessing was defined in the original COLMAP reconstruction coordinate system. In this study, the voxel size was set to 0.02 reconstruction coordinate units. This parameter was used solely to control the sampling density of the network input and should not be confused with the metric voxel size subsequently used for calculating voxel occupancy volume during structural phenotyping.

During the model training stage, SphereCrop was first applied to process the point cloud samples, with a maximum of 50,000 points retained for each input sample to meet the network input requirements. Subsequently, random data augmentation strategies were adopted to improve model generalization capability, including RandomDropout, RandomRotate, RandomScale, RandomFlip, RandomJitter, and ElasticDistortion. In addition, point cloud color information was augmented using ChromaticAutoContrast, ChromaticTranslation, and ChromaticJitter.

The dataset comprised 60 three-dimensional point cloud samples obtained from 12 cultivation trays repeatedly observed over five consecutive acquisition dates, with 12 tray-level samples acquired on each date. In the original model-development workflow, the 12 samples acquired on February 4 (Day 1) were used as a fixed early-stage model-selection set. This acquisition date represented the earliest developmental stage, when many seedlings had not yet emerged and the emerged seedlings exhibited small and incompletely developed stem and leaf structures. Samples from the remaining four acquisition dates were organized into four date-blocked training–validation configurations. In each configuration, samples from three acquisition dates were used for training and samples from one acquisition date were used for validation. The February 4 samples were subsequently used to compare PTV-Seg50, the individual ablation variants, the module combinations, and the complete PTV-SegCo model, and the results obtained on this set contributed to the selection of the final model configuration. Therefore, the February 4 samples constituted a model-selection set rather than an independent or untouched final test set. Furthermore, because the same cultivation trays were repeatedly observed across acquisition dates, this date-based protocol did not ensure independence at the cultivation-tray level. The reported segmentation metrics are therefore interpreted as internal model-development and comparative results under the stated protocol rather than as an unbiased estimate of generalization to previously unseen cultivation trays.

**Figure 2:**
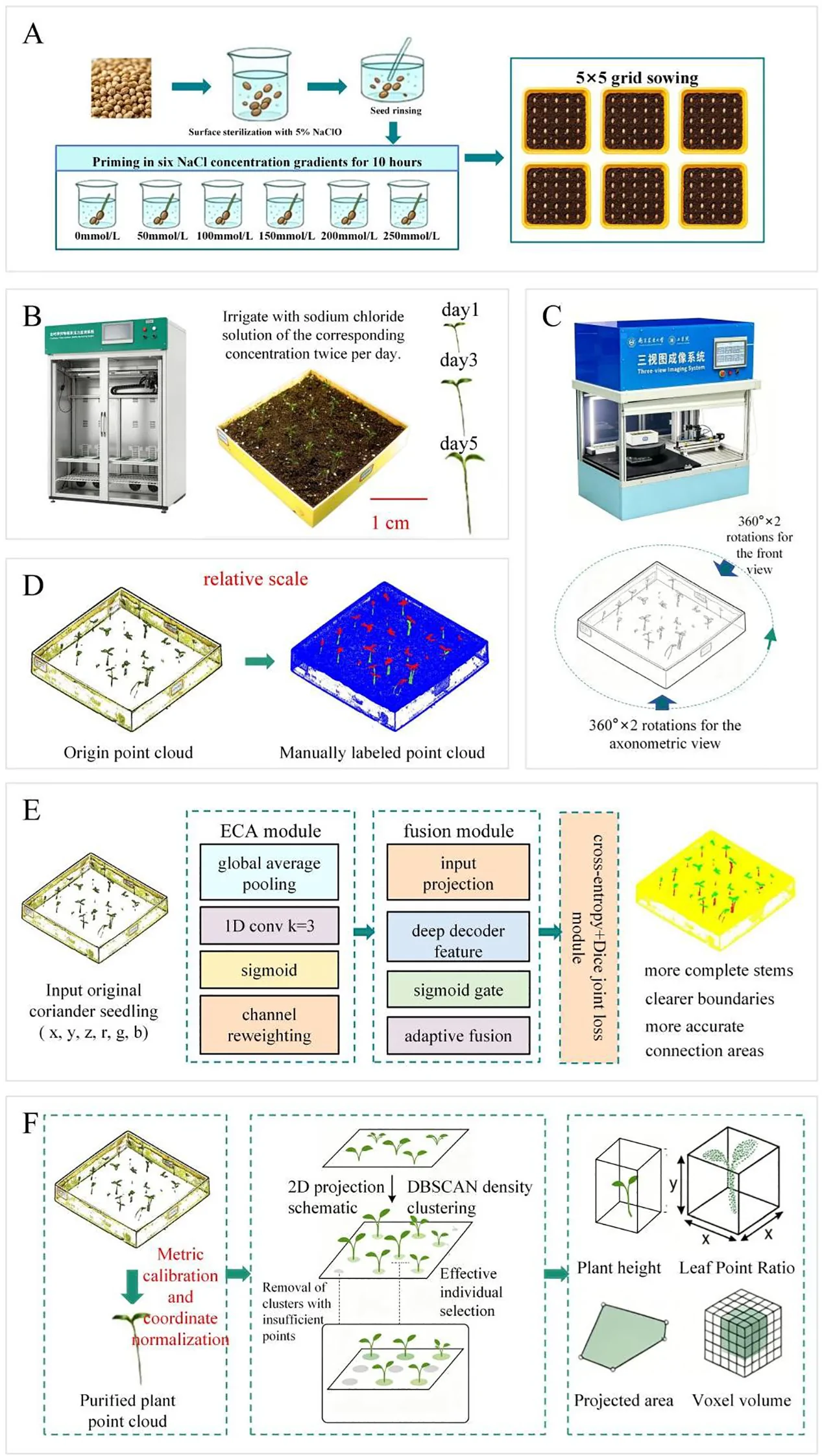
**(A)** Seed pretreatment; **(B)** seedling cultivation; **(C)** multi-view imaging; **(D)** dataset acquisition; **(E)** PTV-segCo model; **(F)** phenotypic trait extraction.

Based on the above three-dimensional point cloud acquisition process, a 3D structural phenotyping workflow for coriander seedlings was further established, as shown in **Figure 2**. The workflow included seed pre-treatment, seedling cultivation, multi-view image acquisition, 3D point cloud reconstruction, manual point cloud annotation, PTV-SegCo semantic segmentation, and structural phenotypic trait extraction. This workflow realized a complete process from raw image data acquisition to automated analysis of three-dimensional structural information.

### 2.3 Design of PTV-SegCo for semantic segmentation of coriander seedling point clouds

**Figure 3:**
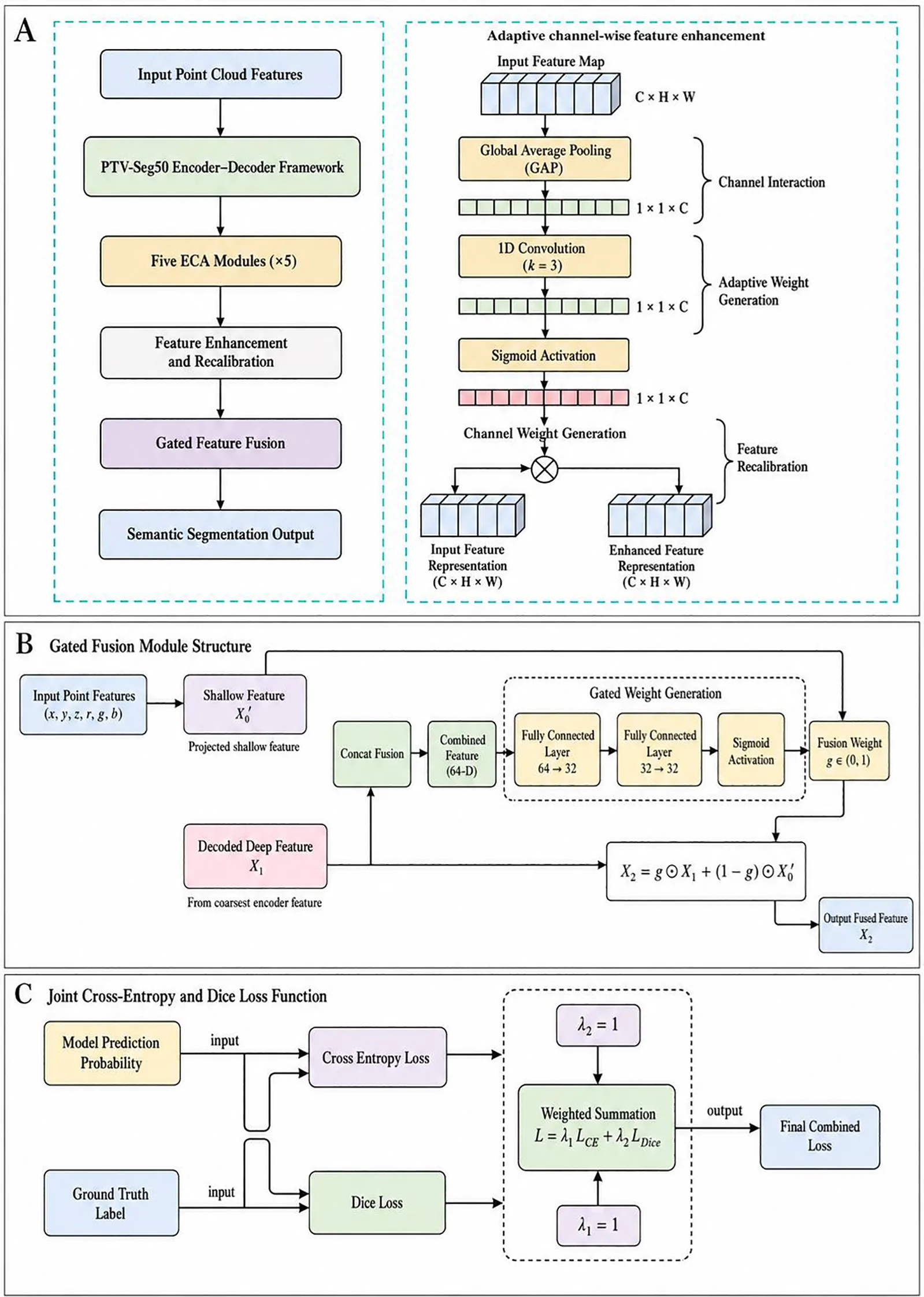
**(A)** Principle of the ECA channel feature enhancement module; **(B)** principle of the gated fusion-based shallow–deep feature fusion module; **(C)** principle of the joint cross-entropy and dice loss function.

To achieve fine-grained semantic segmentation of the pot, stem, and leaf categories in coriander seedling point clouds under salt stress conditions, an improved model, PTV-SegCo, was developed based on PTV-Seg50 in this study. Considering the characteristics of coriander seedling point clouds, including slender leaves, significant differences in organ scales, unclear boundaries between stems and leaves, and imbalanced point distributions among different categories, the original PTV-Seg50 model still has certain limitations in recognizing small-scale organs and segmenting complex boundary regions. Therefore, while maintaining the basic structure of the original Point Transformer encoder–decoder framework (Zhao et al., 2021), this study improved the model from three aspects: feature enhancement, feature fusion, and loss function optimization, according to the structural characteristics of coriander seedling point clouds. Specifically, the Efficient Channel Attention (ECA) mechanism proposed by Wang was introduced to adaptively adjust the weights of different feature channels and enhance the representation of effective features associated with plant organ structures (Wang et al., 2020). Second, a gated shallow–deep feature fusion module was designed to adaptively integrate shallow spatial detail information with deep semantic features, thereby improving the model’s ability to recognize fine stem structures and complex stem–leaf junction regions. Finally, Cross-Entropy Loss was combined with Dice Loss, which optimizes the overlap between predicted and ground-truth regions (Milletari et al., 2016), to alleviate the effects of class imbalance and improve the segmentation capability for small-scale organ regions. The overall network architecture and the design principles of the three improved modules are illustrated in **Figure 3**. The model takes 3D coordinates and color information of points as inputs, where each point contains six-dimensional feature information (x, y, z, r, g, b). The input channel number was set to 6, and the model outputs three semantic categories corresponding to pot, stem, and leaf. Through these improvements, PTV-SegCo can more effectively utilize the spatial structural information and color features of point clouds, thereby improving the segmentation accuracy of fine organs and complex boundary regions in coriander seedling point clouds.

#### 2.3.1 ECA-based channel feature enhancement module

Due to the obvious structural complexity and category-specific characteristics of coriander seedling point cloud data, such as slender leaves, small-scale stem–leaf junction regions, severe local occlusion, and imbalanced feature responses among different semantic categories (i.e., pot, stem, and leaf), the original PTV-Seg50 model still has certain limitations in extracting fine-grained organ structural features. Therefore, an Efficient Channel Attention (ECA) mechanism was introduced into the PTV-Seg50 encoder–decoder framework in this study to enhance the representation capability of key channel features and improve the model’s perception ability for fine structural regions of coriander seedlings.

The ECA module adaptively adjusts the weights of input features by modeling the correlations among different feature channels. For the input feature matrix X, global average pooling (GAP) is first applied to obtain the global representation information of each channel, as shown in Equation 1:

(1) $$z_{c}=\frac{1}{N}\sum_{i=1}^{N}X_{c}(i)$$

Where *N* represents the number of points, *X_c_*(*i*) denotes the feature response of the *i*-th point in the *c*-th channel, and *z_c_* represents the global feature descriptor of the corresponding channel.

Subsequently, a one-dimensional convolution was employed to establish local cross-channel interactions, and the Sigmoid activation function was used to generate channel weights, as shown in Equation 2:

(2) $$\omega =\sigma \left(Conv1D_{k}\left(GAP\left(X\right)\right)\right)$$

Where ω represents the generated channel attention weights, and *Conv*1*D_k_* denotes the one-dimensional convolution operation with a kernel size of *k*. In this study, the kernel size of the one-dimensional convolution in the ECA module was set to 3, enabling the capture of local dependencies among adjacent channels while maintaining relatively low computational complexity.

Finally, the generated channel weights were applied to the original input features to achieve feature recalibration, as shown in Equation 3:

(3) $$X^{\prime }=\omega ⊗X$$

Where *X*′ represents the feature representation after channel enhancement, and ⊗ denotes the channel-wise multiplication operation. Through this process, the model can enhance effective features related to plant organ structures and suppress feature responses with limited contributions to the segmentation task.

As shown in **Figure 3A**, the ECA module mainly consists of four steps: global average pooling, one-dimensional convolution, Sigmoid activation, and channel feature recalibration. Compared with traditional channel attention methods, ECA does not introduce additional fully connected layers, thereby reducing parameter overhead while maintaining effective channel information interaction capability. For key regions in coriander seedling point clouds, such as fine stem structures, leaf boundaries, and stem–leaf junction areas, the ECA module was employed in this study to enhance the network’s ability to represent local detail features, thereby improving feature discrimination among different organ categories.

#### 2.3.2 Gated fusion module for joint shallow and deep feature integration

In point cloud semantic segmentation tasks, shallow features usually contain rich spatial geometric information, such as point positional relationships, local edges, and fine structural details, whereas deep features extracted through multiple network layers possess stronger semantic representation capabilities but may lose some detailed information during continuous feature extraction. For coriander seedling point clouds, fine stem structures, leaf edges, and stem–leaf junction regions are characterized by small scales, complex structures, and susceptibility to occlusion. Therefore, accurate segmentation is difficult to achieve using features from a single level alone. In this study, a Gated Fusion Module was designed to adaptively fuse shallow spatial information from the encoder and deep semantic information from the decoder.

As shown in **Figure 3B**, the module first obtains shallow features and deep features separately. Among them, the shallow feature X0′ mainly preserves local geometric structural information of the point clouds, while the deep feature X1 contains high-level semantic information extracted through network encoding and decoding. To fully exploit the complementary characteristics of the two types of features, the shallow and deep features are first concatenated and fused as shown in Equation 4:

(4) $$X_{c}=Concat\left(X_{0}^{\prime },X_{1}\right)$$

Subsequently, the adaptive fusion weights for different feature sources were calculated through the gated weight generation module, as shown in Equation 5:

(5) $$g=\sigma \left(W_{g}X_{c}+b_{g}\right)$$

Where *g* represents the gating weight, σ denotes the Sigmoid activation function, and *W_g_* and *b_g_* represent learnable parameters, respectively. The gating weights can dynamically adjust the contribution proportions of shallow and deep features in the final feature representation according to the input feature characteristics.

In the implementation, the gating weight generation module consisted of two fully connected layers, transforming the concatenated feature representation from 64 dimensions to 32 dimensions and subsequently generating adaptive fusion representations. This lightweight design enabled adaptive feature weighting while introducing limited additional computational cost.

The final fused feature is expressed as shown in Equation 6:

(6) $$X_{2}=g⊙X_{1}+\left(1-g\right)⊙X_{0}^{\prime }$$

Where ⊙ denotes the element-wise multiplication operation, and *X*_2_ represents the fused feature. Through this mechanism, the network can select more effective information sources according to the structural characteristics of different regions. For regions requiring fine spatial localization, such as leaf edges and fine stems, the model can enhance the contribution of shallow geometric features. For tasks involving organ category discrimination, deep semantic features can be utilized to improve classification capability.

Compared with simple feature concatenation, the gated fusion mechanism can avoid information redundancy caused by directly combining features from different levels and improve the effectiveness of feature fusion through dynamic weight allocation. This module addresses the difficulty of fine organ recognition and the loss of local structural information in coriander seedling point clouds, thereby contributing to improved segmentation accuracy in boundary regions among the pot, stem, and leaf categories.

#### 2.3.3 Joint loss function based on cross-entropy and dice

In three-dimensional point cloud semantic segmentation tasks, the imbalanced distribution of points among different categories is one of the important factors affecting model performance. In this study, coriander seedling point clouds contain three semantic categories: pot, stem, and leaf, with significant differences in their spatial proportions. Among them, the pot category generally exhibits a relatively complete continuous structure, whereas plant organ regions such as stems and leaves may contain fewer effective points due to morphological characteristics and occlusion effects. Therefore, using only the conventional Cross-Entropy Loss may cause the model to focus more on categories with larger proportions, thereby reducing its recognition capability for small-scale organ regions. To address this issue, a combined loss function integrating Cross-Entropy Loss and Dice Loss was constructed in this study, and its overall calculation process is illustrated in **Figure 3C**.

To further quantitatively analyze the degree of class imbalance, the numbers and proportions of points belonging to different semantic categories were statistically calculated from the 60 manually annotated three-dimensional point cloud samples. Detailed statistical results are presented in **Supplementary Table 1**. The overall category distribution is shown in **Table 3**. The pot category contained a total of 65,548,031 points, accounting for 96.73% of all annotated point-cloud points, while the stem and leaf categories accounted for only 1.30% and 1.97%, respectively. These results demonstrate a substantial imbalance in data distribution between the background category and plant organ categories. In particular, the stem category was more susceptible to the influence of class imbalance because of its small structural scale, accounting for only 1.30% of all annotated points in the dataset. Therefore, Dice Loss was introduced during model training to improve the model’s attention to minority category regions. Therefore, Dice Loss was introduced during model training to improve the model’s attention to minority category regions.

**Table 3:** Overall semantic class distribution of the complete coriander seedling point-cloud dataset.

Semantic class	Total points	Percentage (%)
Pot	65,548,031	96.73
Stem	878,428	1.30
Leaf	1,337,482	1.97

To improve the segmentation capability of the model for different category regions, a combined loss function integrating Cross-Entropy Loss and Dice Loss was adopted for model optimization, as shown in Equation 7:

(7) $$L=\lambda_{1}L_{CE}+\lambda_{2}L_{Dice}$$

Where *L_CE_* represents the Cross-Entropy Loss, which is used to measure the difference between the predicted class probability distribution and the ground-truth labels; *L_Dice_* represents the Dice Loss, which is used to evaluate the overlap between the predicted regions and the ground-truth regions; λ_1_ and λ_2_ denote the weighting coefficients of the two loss functions.

The Cross-Entropy Loss is defined as shown in Equation 8:

(8) $$L_{CE}=-\frac{1}{N}\sum_{i=1}^{N}\sum_{c=1}^{C}y_{ic}\log(p_{ic})$$

Where *N* represents the number of input points, *C* represents the number of categories, *y_ic_* denotes the ground-truth label indicating whether the *i*-th point belongs to category *c*, and *p_ic_* represents the probability predicted by the model that the point belongs to category *c*.

The Dice Loss is defined as shown in Equation 9:

(9) $$L_{Dice}=1-\frac{2\sum_{c=1}^{C}p_{c}y_{c}+\epsilon }{\sum_{c=1}^{C}p_{c}+\sum_{c=1}^{C}y_{c}+\epsilon }$$

Where *p_c_* and *y_c_* represent the prediction results and ground-truth labels for category *c*, respectively, and *ϵ* is a smoothing term introduced to prevent division by zero.

During model training, the two loss functions were assigned equal weights for joint optimization, namely: $\;\lambda_{1}=\lambda_{2}=1$By combining the category discrimination capability of Cross-Entropy Loss and the region overlap optimization capability of Dice Loss, the joint loss function improves the model’s ability to learn boundaries among different organ categories. In particular, it contributes to improving the segmentation performance of fine stem structures, leaf edges, and regions affected by class imbalance.

#### 2.3.4 Comparison of computational complexity and inference efficiency between PTV-Seg50 and PTV-SegCo models

To further evaluate the applicability of the proposed PTV-SegCo model in practical three-dimensional plant phenotyping tasks, the computational complexity and operational efficiency of the baseline model PTV-Seg50 and the improved model PTV-SegCo were compared under the same hardware and software environment. The specific evaluation metrics included the number of Parameters, Training time, and average Inference time for a single point cloud sample. Both models were evaluated using the same data partitioning strategy, number of training epochs, and experimental configurations. The experimental results are presented in **Table 4**.

**Table 4:** Computational complexity and efficiency comparison between PTV-Seg50 and PTV-SegCo.

Model	Parameters(M)	Training time (h)	Inference time (s/sample)
PTV-Seg50	7.77	2.45	93.4
PTV-SegCo	7.77	2.33	83.2

All measurements were obtained under the same hardware and software environment. The inference time represents the average processing time per point cloud sample.

The results in **Table 4** show that the PTV-SegCo model contains 7.77 M parameters, which is essentially the same as the baseline model PTV-Seg50 (7.77 M). This indicates that the introduced ECA channel attention module and gated shallow–deep feature fusion module did not significantly increase the model size. In terms of training time, PTV-SegCo required approximately 2.33 h, which was comparable to PTV-Seg50 (2.45 h). Regarding inference efficiency, PTV-SegCo required an average of 83.2 s to process a single point cloud sample, while PTV-Seg50 required 93.4 s, indicating that both models maintained similar inference efficiency.

Overall, after incorporating feature enhancement and fusion strategies, PTV-SegCo introduced only negligible changes in model parameters while maintaining computational costs comparable to the baseline model. Under the present experimental configuration, PTV-SegCo showed numerically higher segmentation metrics than its direct backbone PTV-Seg50 while introducing negligible changes in model parameters and comparable computational costs. These results suggest that the proposed task-specific modifications did not substantially increase the computational burden relative to PTV-Seg50. However, the computational efficiency and segmentation performance of PTV-SegCo relative to other point-cloud segmentation architectures were not evaluated in this study.

### 2.4 Three-dimensional structural phenotyping based on semantic point clouds

**Figure 4:**
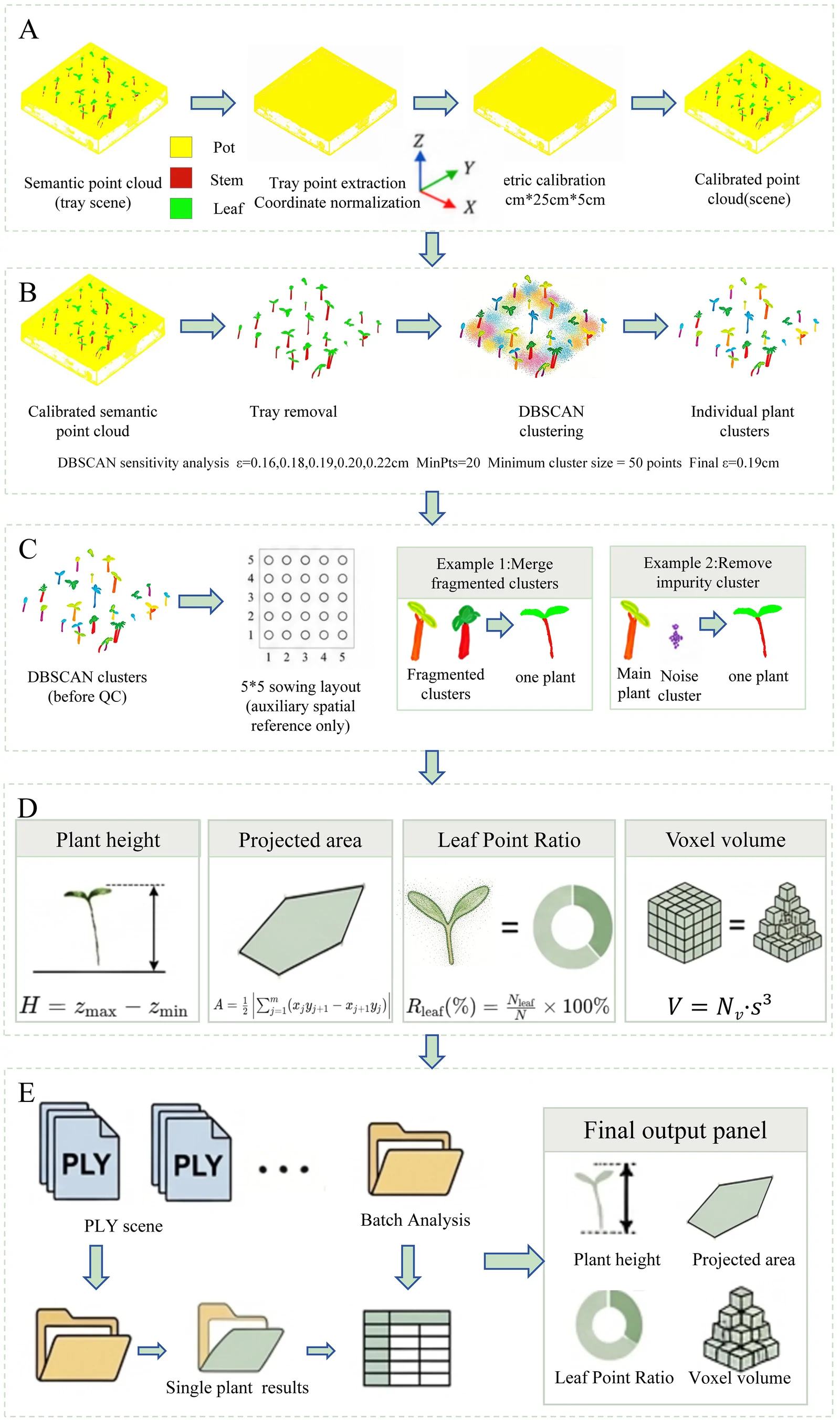
**(A)** Metric calibration and coordinate normalization. **(B)** Individual-plant separation and DBSCAN parameter sensitivity. **(C)** Individual-plant quality control and manual correction. **(D)** Structural phenotypic trait calculation. **(E)** Batch processing and output.

Following organ-level semantic segmentation, a separate post-processing workflow was established for quantitative three-dimensional structural phenotyping. As described in Section 2.2, the point clouds used for model development and semantic segmentation retained the original relative coordinate system generated by COLMAP and therefore did not directly represent physical measurement units. Accordingly, metric calibration was performed only after semantic segmentation and before quantitative phenotypic analysis. The subsequent workflow consisted of metric calibration and coordinate normalization, semantic filtering and individual-plant separation, clustering quality control and manual correction, structural phenotypic trait calculation, and batch output. The calibration and post-processing procedures modified only the spatial reference used for quantitative measurement and did not alter the semantic labels or the previously completed model training and evaluation. The complete structural phenotyping workflow is illustrated in **Figure 4**.

#### 2.4.1 Metric calibration and coordinate normalization

Because the COLMAP reconstruction did not provide an absolute physical scale, metric calibration and coordinate normalization were performed before quantitative structural phenotyping, as illustrated in **Figure 4A**. The acrylic cultivation tray, with known physical dimensions of 25 cm × 25 cm × 5 cm, was used as an external geometric reference for each reconstructed scene. Points belonging to the pot category were first extracted from each semantic point cloud to define the tray geometry. Principal component analysis (PCA) was then used to estimate the dominant orientation of the tray and align its surface normal with the Z-axis. Subsequently, a minimum-area rectangle was fitted to the horizontal projection of the tray, and its two principal edges were aligned with the X- and Y-axes.

After orientation normalization, the reconstructed horizontal dimensions of the tray were compared with the known physical side length of 25 cm to determine a scene-specific isotropic scale factor. The same scale factor was uniformly applied to the X-, Y-, and Z-coordinates, thereby converting the reconstructed coordinates to centimeters while preserving the original three-dimensional geometric proportions of the scene. The upper surface of the cultivation tray was subsequently defined as the vertical reference plane. The known tray height of 5 cm was used only as an auxiliary plausibility check of the calibrated vertical scale and was not used as a separate Z-axis scaling constraint, thereby avoiding anisotropic deformation of plant geometry.

This calibration and orientation-normalization procedure was applied independently to all reconstructed scenes before individual-plant separation and structural phenotypic calculation. Consequently, the dimensional structural descriptors reported in this study, including plant height, projected area, and voxel occupancy volume, were calculated from the metric-calibrated point clouds and expressed in cm, cm^2^, and cm^3^, respectively.

#### 2.4.2 Individual plant separation and DBSCAN parameter sensitivity

After metric calibration and coordinate normalization, points belonging to the pot category were removed from the semantic point clouds, whereas stem- and leaf-category points were retained for individual-plant analysis. As illustrated in **Figure 4B**, the remaining plant points were projected onto the calibrated XY plane, and DBSCAN (Density-Based Spatial Clustering of Applications with Noise) was applied to separate spatially independent plant instances.

Because the DBSCAN neighborhood radius directly affects the connectivity of spatially discontinuous plant structures, a parameter-sensitivity analysis was conducted before the final batch extraction. Five candidate neighborhood radii, ϵ = 0.16, 0.18, 0.19, 0.20, and 0.22 cm, were evaluated while MinPts was fixed at 20. Clusters containing fewer than 50 points were regarded as invalid small components and excluded from subsequent phenotypic calculation.

Twenty representative scenes were selected for the sensitivity analysis. These scenes included samples that exhibited relatively unusual preliminary plant counts and also covered different NaCl treatments, acquisition dates, and plant densities. For each candidate ϵ value, the number of detected plant clusters and the corresponding point-to-cluster assignments were recorded. Cluster-specific pseudo-colored point clouds were additionally generated and visually inspected to evaluate clustering quality. Over-segmentation was defined as the separation of a single biological plant into two or more disconnected clusters, whereas under-segmentation was defined as the merging of two or more spatially distinct seedlings into a single cluster. Missed plants and false clusters generated by small disconnected fragments or non-plant components were also checked during manual review. The scene-level cluster counts obtained under the five candidate ϵ values and the corresponding manual interpretations are provided in **Supplementary Table 2**.

Based on the parameter-sensitivity analysis and visual inspection, ϵ = 0.19 cm with MinPts = 20 was selected for the subsequent batch individual-plant separation. Final clustering outputs were subjected to additional quality control and manual correction as described in Section 2.4.3.

#### 2.4.3 Individual-plant quality control and manual correction

Following DBSCAN-based individual-plant separation, the clustering results of all scenes were subjected to quality control before structural phenotypic calculation, as illustrated in **Figure 4C**. Cluster-specific pseudo-colored point clouds were visually inspected together with the corresponding calibrated scene point clouds to identify potential separation errors. The predefined 5 × 5 sowing layout was used only as an auxiliary spatial reference during this inspection to help identify implausible duplicate detections, fragmented plant structures, and clusters inconsistent with the expected planting positions.

Particular attention was paid to over-segmentation, in which spatially disconnected parts of the same biological plant were assigned to different clusters, and to false detections caused by small impurity or noise components. When two disconnected clusters were manually confirmed to represent parts of the same biological plant based on their spatial proximity, morphological continuity, and relationship to the corresponding sowing position, their point sets were merged before phenotypic calculation. Conversely, clusters that were confirmed to represent non-plant impurity or noise components were removed. Spatially adjacent but morphologically distinct complete seedlings were retained as separate plant instances. The manually confirmed correction records are summarized in **Supplementary Table 3**.

The 5 × 5 sowing layout was not used to force one-to-one correspondence of individual plants across acquisition dates. Because seedling emergence, occlusion, reconstruction completeness, and detectable plant numbers varied among acquisition dates, reliable longitudinal identity assignment of every individual seedling could not be guaranteed. Therefore, no automatic cross-date plant matching was used in the final phenotypic analysis, and individual seedlings were not treated as longitudinal repeated-measurement units. Instead, each acquisition scene was quality-controlled independently, and the resulting QC-valid individual-plant measurements were subsequently summarized at the cultivation-tray-by-acquisition-time level for statistical analysis.

#### 2.4.4 Structural phenotypic trait calculation

Following metric calibration, individual-plant separation, and quality control, four reconstruction-derived structural descriptors were obtained. Plant height, projected area, and voxel occupancy volume were calculated from the QC-valid individual-plant point clouds, whereas the leaf point ratio was calculated at the reconstructed-scene level from the relative numbers of semantic-class points. As illustrated in **Figure 4D**, these descriptors characterize different aspects of the reconstructed three-dimensional plant structure, including vertical extent, horizontal spatial coverage, three-dimensional point-cloud occupancy, and the relative distribution of leaf-category points. All dimensional descriptors were calculated in the calibrated metric coordinate system described in Section 2.4.1.

Plant height was used to characterize the vertical extent of each coriander seedling. As schematically illustrated in the plant-height component of **Figure 4D**, plant height was calculated as the difference between the maximum and minimum vertical coordinates of the verified individual-plant point cloud, and its calculation process is shown in Equation 10:

(10) $$H=z_{\max}-z_{\min}$$

Where *z*_max_ and *z*_min_ denote the maximum and minimum Z-coordinates of the individual-plant point cloud, respectively. Because the calculation was performed after metric calibration and coordinate normalization, plant height was expressed in centimeters (cm).

Projected area was used to characterize the horizontal spatial coverage of each plant. As shown in the projected-area component of **Figure 4D**, the three-dimensional plant point cloud was first projected onto the calibrated XY plane, after which a two-dimensional convex hull was constructed from the projected points. The projected area enclosed by the convex hull was calculated using Equation 11:

(11) $$A=\frac{1}{2}\left|\sum_{i=1}^{m\;}(x_{i}y_{i+1}-x_{i+1}y_{i})\right|$$

Where *x_i_* and *y_i_* represent the coordinates of the projected points, and n represents the number of boundary points of the two-dimensional convex hull.

To describe the spatial occupancy range of plant point clouds in three-dimensional space, a voxelization method was further applied to calculate the voxel occupancy volume. Assuming that the voxel side length is s and the number of valid occupied voxels is *N_v_*, the voxel occupancy volume is calculated as shown in Equation 12:

(12) $$V=N_{v}\times s^{3}$$

where *N_v_* denotes the number of occupied voxels and s denotes the voxel side length. In the final structural phenotyping analysis, s was fixed at 0.062 cm; therefore, voxel occupancy volume was expressed in cubic centimeters (cm^3^). This descriptor quantifies the spatial occupancy of the reconstructed point cloud rather than the true physical volume of plant tissues. Accordingly, it was not interpreted as a direct measure of biomass, dry-matter accumulation, or tissue volume.

Furthermore, to describe the relative contribution of leaf-category points within each reconstructed scene, a scene-level leaf point ratio was calculated as shown in Equation 13:

(13) $${\text{R}}_{\text{leaf}}=({\text{N}}_{\text{leaf}}/{\text{N}}_{\text{total}})\times 100\%$$

where N_leaf_ represents the number of points assigned to the leaf category in the reconstructed scene, and N_total_ represents the total number of points belonging to the three modeled semantic categories (pot, stem, and leaf) in the same scene. Thus, N_total_=N_pot_+N_stem_+N_leaf_. The leaf point ratio represents the proportion of leaf-category points in the reconstructed semantic point cloud and was used as a descriptive indicator of changes in the relative leaf-related point distribution.

Each individual-plant record contained the corresponding NaCl treatment, cultivation tray, acquisition date, within-scene plant identifier, plant height, projected area, and voxel occupancy volume. The scene-level leaf point ratio was calculated and stored separately for each reconstructed cultivation-tray scene. For treatment-level statistical inference, these measurements were subsequently aggregated within each cultivation tray at each acquisition time, as described in Section 2.5. Individual seedlings were therefore not treated as independent treatment replicates or as longitudinal repeated-measurement units across acquisition dates.

#### 2.4.5 Independent validation of plant height and projected area

To independently evaluate the measurement accuracy of the reconstruction-derived structural phenotypic descriptors, plant height and projected area were compared with manually obtained reference measurements. The validation dataset comprised 30 paired treatment-by-time observations covering all six NaCl concentrations and five acquisition times. For each NaCl treatment and acquisition time, the manually obtained reference value was paired with the corresponding point-cloud-derived value for agreement analysis.

Manual plant height was measured directly using a ruler and recorded to the nearest 1 mm. For projected area, an independent manual approximation was obtained by representing the horizontal plant projection as an equivalent rectangle. The maximum projected length and maximum projected width of the whole plant were each measured five times using a ruler with a 1-mm resolution. The five measurements were averaged separately to obtain the mean projected length and mean projected width, and the manually approximated projected area was calculated as the product of these two mean dimensions. The resulting reference values were expressed in cm for plant height and cm^2^ for projected area. Because the manual projected-area measurement approximated the horizontal plant projection using an equivalent rectangle rather than tracing the exact plant boundary, it was regarded as an independent practical reference for agreement assessment rather than an exact ground-truth area.

Agreement between the point-cloud-derived and manual reference measurements was quantified using mean bias, mean absolute error (MAE), root mean square error (RMSE), and the coefficient of determination (R^2). Bias was defined as the point-cloud-derived value minus the corresponding manual reference value, such that a negative bias indicated underestimation by the point-cloud-derived measurement. Linear regression was additionally performed between the manual reference measurements and the corresponding point-cloud-derived values, and the fitted regression line was compared with the 1:1 reference line to visually assess measurement agreement. The quantitative validation results are reported in Section 3.4.3.

### 2.5 Statistical analysis

The cultivation tray was considered the independent experimental unit for evaluating the effects of NaCl treatment. Two independent cultivation trays were included for each NaCl concentration, whereas individual seedlings within the same cultivation tray were treated as subsamples rather than independent biological replicates. Following individual-plant separation and quality control, measurements of each structural phenotypic descriptor were averaged across all QC-valid seedlings within each cultivation tray at each acquisition time. Consequently, one tray-level observation was obtained for each tray-by-time combination. The statistical analysis therefore comprised 12 independent cultivation trays repeatedly observed at five acquisition times, yielding 60 tray-level observations for each structural phenotypic descriptor.

The effects of NaCl concentration and acquisition time on the structural phenotypic descriptors were evaluated using linear mixed-effects models (LMMs) (Laird and Ware, 1982). NaCl concentration, acquisition time, and their interaction were specified as fixed effects, whereas cultivation tray nested within NaCl treatment was included as a random intercept. Acquisition time was treated as a categorical factor. Repeated observations obtained from the same cultivation tray across the five acquisition times were therefore treated as repeated measurements of the same experimental unit, with within-tray correlation accounted for by the shared tray-specific random intercept. No additional residual correlation structure was imposed. The model was expressed as shown in Equation 14:

(14) $${\hat{Y}}_{ijt}=\mu +\alpha_{i}+\tau_{t}+\left(\alpha \tau \right)it+bj\left(i\right)+\epsilon_{ijt}$$

Where $\hat{\text{Y}}\text{ijt}$ represents the tray-level mean of a structural phenotypic descriptor for the j-th cultivation tray under the i-th NaCl treatment at acquisition time t; μ represents the overall mean; α_i_ represents the fixed effect of NaCl treatment; τ_t_ represents the fixed effect of acquisition time; (ατ)it represents the interaction between NaCl treatment and acquisition time; b_j(i)_ represents the random intercept associated with cultivation tray nested within NaCl treatment; and ϵ_ijt_ represents the residual error. Individual seedlings within each cultivation tray contributed only to the calculation of the corresponding tray-level mean and were not treated as independent experimental replicates for inference on NaCl treatment effects.

The LMMs were fitted by restricted maximum likelihood (REML) using the MixedLM implementation in the statsmodels package (version 0.14.6) in Python, with the BFGS optimization algorithm. Preliminary residual diagnostics indicated substantial departure from normality for projected area on the original scale; therefore, projected area was natural-log transformed before model fitting. Plant height and voxel occupancy volume were analyzed on their original scales. Leaf point ratio was treated as a descriptive reconstruction-derived structural descriptor and was not included in the inferential mixed-effects analysis. Statistical significance was assessed at p< 0.05.

No complete tray-by-time observations were missing from the final quality-controlled dataset. Individual seedlings or point-cloud components excluded during the quality-control procedure were not assigned zero values and were not imputed. Instead, the tray-level mean at each acquisition time was calculated using all QC-valid seedlings available within the corresponding cultivation tray. Thus, differences in the number of valid individual seedlings among tray-by-time combinations did not increase the number of independent experimental replicates used for treatment-level inference.

**Figure 5:**
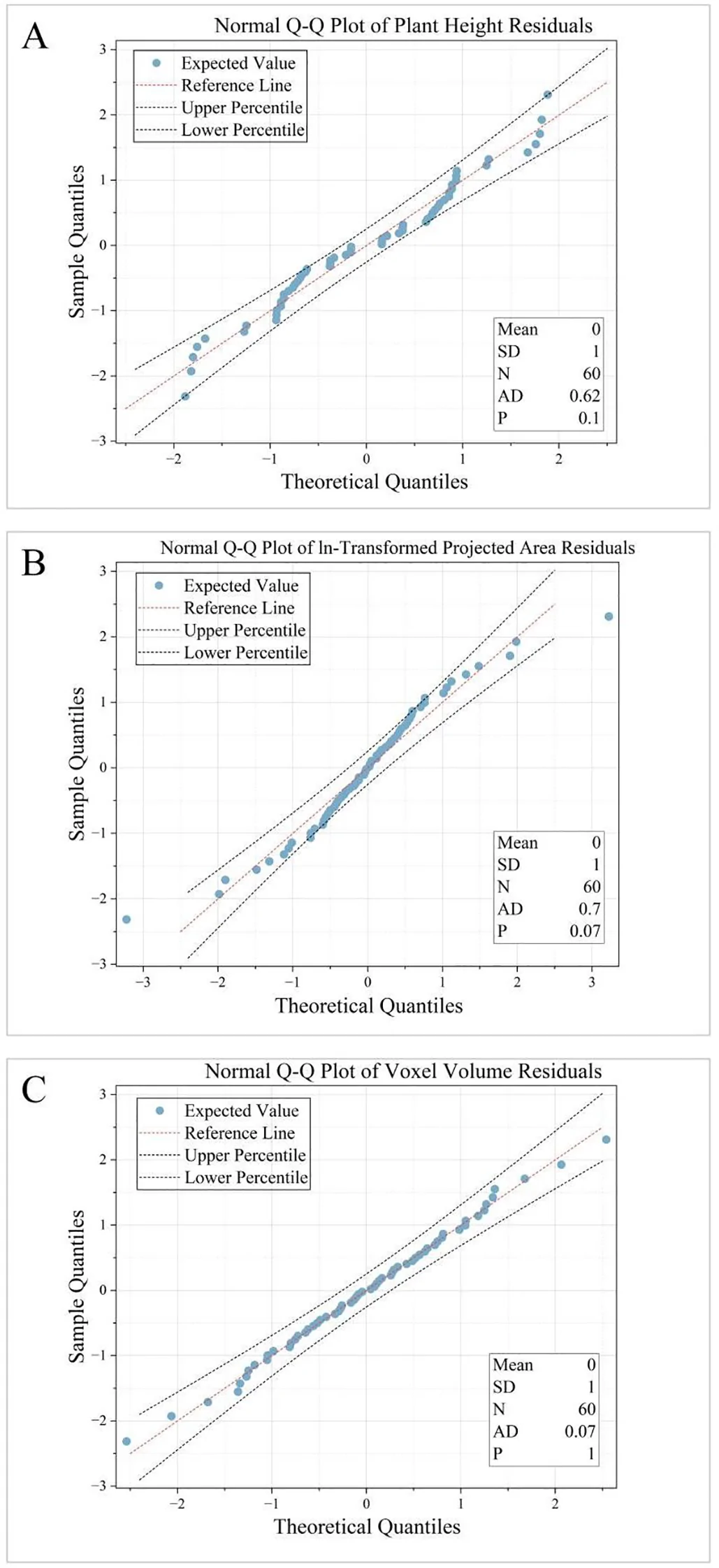
Normal Q-Q plots of standardized residuals from the tray-level linear mixed-effects models for three structural phenotypic descriptors: **(A)** plant height, **(B)** natural-log-transformed projected area, and **(C)** voxel occupancy volume. The red dashed line represents the theoretical normal reference line, and the black dashed lines indicate the pointwise 95% reference envelope. N denotes the number of tray-by-time observations included in each model, AD denotes the Anderson-Darling statistic, and P denotes the p-value from the Shapiro-Wilk normality test.

Model convergence and residual distributions were examined before statistical inference. Residual normality was evaluated using normal Q-Q plots together with the Anderson-Darling and Shapiro-Wilk diagnostics. As shown in **Figures 5A–C**, the standardized residuals of plant height, natural-log-transformed projected area, and voxel occupancy volume generally followed the theoretical normal reference line and remained largely within the 95% reference envelopes. The Anderson-Darling statistics were 0.622, 0.695, and 0.074 for plant height, natural-log-transformed projected area, and voxel occupancy volume, respectively, all below the corresponding 5% critical value of 0.742. The corresponding Shapiro–Wilk tests yielded p=0.095, p=0.064, and p>0.99, respectively. These diagnostic results indicated no substantial departure from residual normality for the three dimensional structural descriptors after transformation of projected area. Because only two independent cultivation trays were available for each NaCl treatment, statistical inference regarding treatment effects was interpreted cautiously and was restricted to the experimental conditions investigated in this study.

For omnibus inference on the fixed effects, NaCl concentration and acquisition time were represented using sum-to-zero contrasts, and the overall effects of NaCl concentration, acquisition time, and their interaction were evaluated using joint Wald χ^2^ tests of the corresponding fixed-effect terms.

## 3 Results

### 3.1 Runtime environment

The experiments in this study were conducted under the Ubuntu 22.04.5 LTS operating system, and both model training and model evaluation were implemented based on the Pointcept point cloud deep learning framework. The detailed experimental environment configuration is presented in **Table 5**. To improve training efficiency and reduce GPU memory consumption, automatic mixed-precision computation was enabled during the training process. All comparison experiments and ablation experiments were conducted under the same hardware and software conditions to ensure the comparability of experimental results.

**Table 5:** Experimental simulation system configuration.

Type	Configuration
Operating System	Ubuntu 22.04.5 LTS
CPU	Intel Xeon CPU E5–2697 v4 @ 2.30 GHz
RAM	64 GB
GPU	NVIDIA GeForce RTX 3090
Video Memory	24 GB
Python	3.10.18
Deep Learning Framework	PyTorch 2.0.0
CUDA	12.1
cuDNN	8902
Development Framework	Pointcept

### 3.2 Dataset and experimental settings

The dataset used in this study was derived from three-dimensional point cloud data of coriander seedlings under different salt stress treatments. Through continuous multi-time-point acquisition and three-dimensional reconstruction, a total of 60 valid point cloud samples with manual annotations were obtained. These 60 samples were collected from five consecutive acquisition dates, with 12 tray-level point-cloud samples acquired on each date. Each point cloud sample was manually annotated and divided into three categories: pot, stem, and leaf. Points belonging to invalid regions outside the target categories were uniformly assigned as ignored labels during model training.

To meet the data input requirements of the Pointcept point cloud deep learning framework, all point cloud samples were uniformly organized and formatted before training. Each point contained six-dimensional input features, including three-dimensional spatial coordinates and color information, namely (x, y, z, r, g, b). The number of output categories was set to 3, corresponding to the three semantic categories of pot, stem, and leaf. During model training, validation, and model-selection evaluation, the same point cloud preprocessing procedures and input feature formats were applied to ensure consistency of point cloud feature representations across different stages.

The dataset consisted of repeated observations of 12 cultivation trays over five consecutive acquisition dates. In the original model-development protocol, the 12 samples acquired on February 4 (Day 1) were excluded from gradient-based model training and checkpoint selection and were used as a fixed early-stage model-selection set. This date corresponded to the earliest and most structurally challenging developmental stage, when many seedlings had not yet emerged and the emerged seedlings possessed small and incompletely developed organs. Samples from the remaining four acquisition dates were arranged into four date-blocked training–validation configurations. In each configuration, three acquisition dates, comprising 36 samples, were used for training, while one acquisition date, comprising 12 samples, was used for validation. The same 12 February 4 samples were then used to compare the baseline model, ablation variants, module combinations, and the complete PTV-SegCo model. Because the results obtained on this set contributed to final architecture selection, it did not constitute an untouched final test set. Moreover, because observations from the same cultivation trays occurred across the date-based subsets, the protocol did not provide tray-level independence. The partition and model-development protocol are summarized in **Table 6**.

**Table 6:** Original date-blocked model-development protocol.

Fold	Training set	Validation set	Fixed early-stage model-selection set
Fold 1	Day 2,3,4 (36 samples)	Day 5 (12 samples)	Day 1 (12 samples)
Fold 2	Day 2,3,5 (36 samples)	Day 4 (12 samples)	Day 1 (12 samples)
Fold 3	Day 2,4,5 (36 samples)	Day 3 (12 samples)	Day 1 (12 samples)
Fold 4	Day 3,4,5 (36 samples)	Day 2 (12 samples)	Day 1 (12 samples)

The February 4 (Day 1) samples were excluded from gradient-based training and validation but were used to compare model variants and determine the final PTV-SegCo configuration. They therefore constituted a model-selection set rather than an untouched final test set. Because the same cultivation trays were repeatedly observed across acquisition dates, the subsets were not independent at the cultivation-tray level.

All comparison experiments and ablation experiments adopted the same data partitioning strategy to ensure fairness and comparability among experimental results. During model training, the AdamW optimizer was used, with an initial learning rate of 0.001 and a weight decay coefficient of 0.05. The model was trained for a total of 100 epochs, and the OneCycleLR strategy was employed for dynamic learning rate adjustment. This study further provides complete experimental configurations and implementation details, including data organization, point cloud preprocessing procedures, model input formats, and training parameter settings, to improve the reproducibility of the proposed method.

### 3.3 Ablation experiments

To verify the contribution of each improved module in the PTV-SegCo model to the semantic segmentation performance of coriander seedling point clouds, ablation experiments were conducted in this study. The independent effects and combined effects of the Efficient Channel Attention (ECA) module, Gated Fusion module, and Cross-Entropy–Dice joint loss function (CE-Dice Loss) were analyzed separately. All ablation variants were trained using the same four date-blocked training–validation configurations described in Section 2.2 and were evaluated on the fixed February 4 early-stage model-selection set. The results obtained on this set were used to compare the individual modules and their combinations and to determine the final PTV-SegCo configuration. Therefore, the values reported in the ablation analysis represent internal model-selection results rather than performance on an independent or untouched final test set. All models used the same preprocessing procedures, training parameters, and evaluation metrics to enable controlled comparisons under the original protocol.

The performance variations of models with different combinations of improvement modules are shown in **Table 7**. Using the baseline model PTV-Seg50 as the reference, its mIoU, mAcc, and stem IoU were 83.77%, 88.87%, and 66.64%, respectively. After independently introducing the ECA module, the model mIoU increased to 86.20%, representing an improvement of 2.43 percentage points compared with the baseline model. Meanwhile, stem IoU increased from 66.64% to 71.68%, with an improvement of 5.04 percentage points. These results indicate that the ECA module enhances the network’s ability to extract fine organ structural features and improves recognition performance for fine stem regions. After incorporating Dice Loss, the model mIoU further increased to 86.43%, stem IoU reached 71.65%, and leaf IoU increased from 84.95% to 87.94%. This result demonstrates that Dice Loss can alleviate the class imbalance problem, enabling the model to pay more attention to plant organ regions with fewer points and improving segmentation balance among different categories. After independently adding the Gated Fusion module, the model achieved an mIoU of 86.38%, improving by 2.61 percentage points compared with PTV-Seg50, while stem IoU increased to 71.60%. This result indicates that by integrating shallow spatial structural information and deep semantic information, the model can more effectively utilize local geometric features and high-level category information from point clouds, thereby improving segmentation performance in complex boundary regions.

**Table 7:** Ablation experiment results.

Index	allAcc	mAcc	mIoU	IoU-pot	IoU-stem	IoU-leaf
PTV-Seg50	0.99552	0.8887	0.8377	0.9970	0.6664	0.8495
+ECA	0.9951	0.9082	0.8620	0.9970	0.7168	0.8720
+dice	0.9951	0.9123	0.8643	0.9969	0.7165	0.8794
+fusion	0.9951	0.9068	0.8638	0.9970	0.7160	0.8782
+ECA+dice	0.9953	0.9137	0.8690	0.9971	0.7267	0.8831
+ECA+fusion	0.9952	0.9089	0.8648	0.9971	0.7180	0.8793
+dice+fusion	0.9952	0.9091	0.8650	0.9971	0.7190	0.8790
PTV-SegCo	0.9961	0.9305	0.8905	0.9979	0.7734	0.9002

The February 4 samples were used to compare the ablation variants and select the final model architecture. The reported values therefore represent internal model-development results rather than independent final test performance.

Further analysis of the effects of different module combinations revealed that multi-module integration produced more significant performance improvements. The combination of ECA and Dice Loss achieved an mIoU of 86.90% and increased stem IoU to 72.67%, representing improvements of 3.13 and 6.03 percentage points compared with the baseline model, respectively. The combination of ECA and Gated Fusion achieved an mIoU of 86.48%, while the combination of Dice Loss and Gated Fusion achieved an mIoU of 86.50%. These results indicate that different optimization strategies address different limitations, including insufficient feature representation, inadequate spatial information fusion, and class imbalance, and exhibit complementary effects. Among the model configurations evaluated on the fixed February 4 model-selection set, the configuration integrating ECA, Gated Fusion, and CE-Dice Loss produced the highest mAcc, mIoU, stem IoU, and leaf IoU values. The allAcc, mAcc, and mIoU of PTV-SegCo reached 99.61%, 93.05%, and 89.05%, respectively. Compared with the baseline PTV-Seg50 model, mAcc increased by 4.18 percentage points and mIoU increased by 5.28 percentage points. Meanwhile, stem IoU increased from 66.64% to 77.34%, representing an improvement of 10.70 percentage points, and leaf IoU increased from 84.95% to 90.02%, representing an improvement of 5.07 percentage points. These internal comparisons indicate that the combined configuration was associated with numerically improved recognition of fine stem structures and leaf boundary regions under the original model-development protocol.

**Figure 6:**
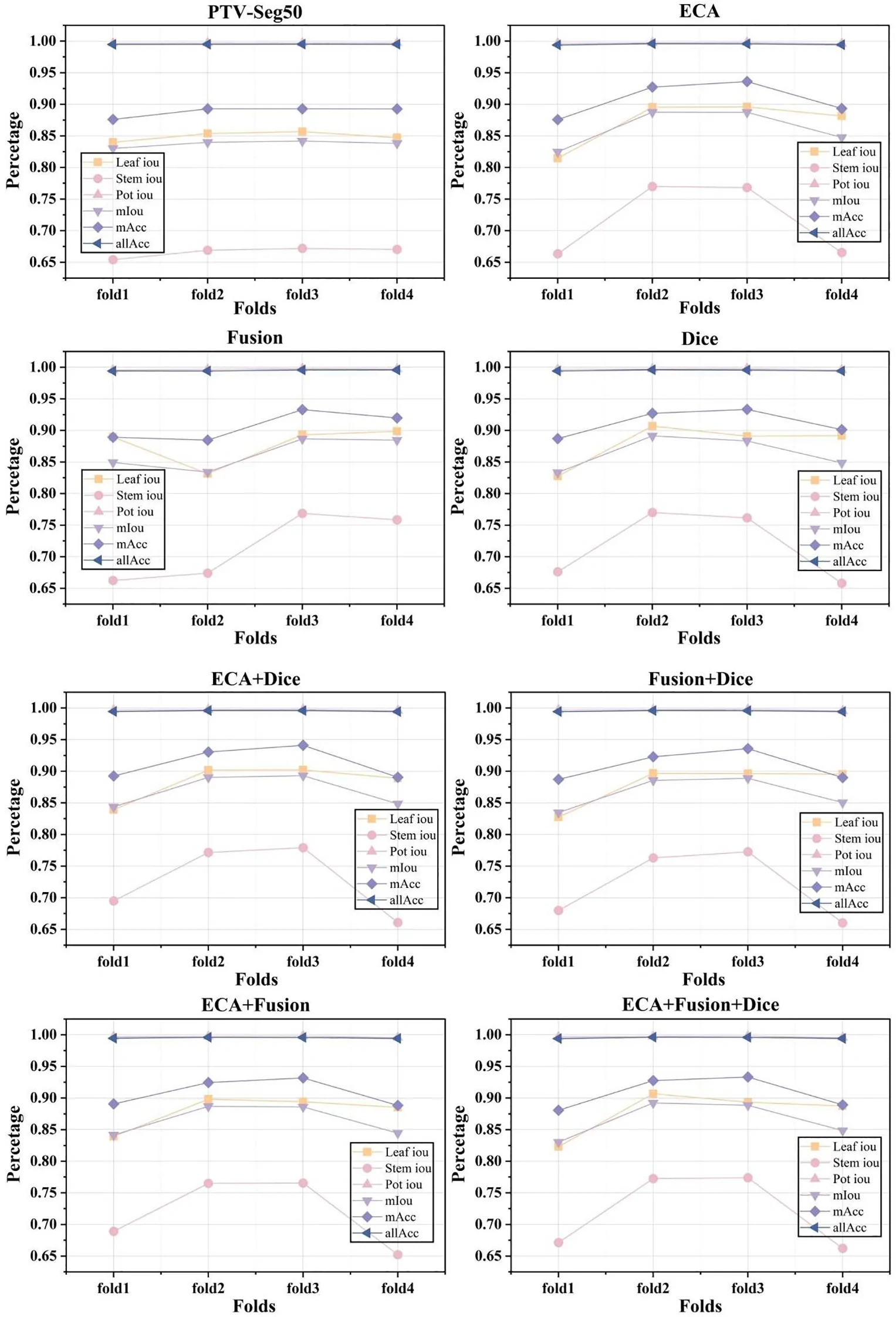
Performance across four date-blocked training–validation configurations.

The performance of PTV-Seg50 and PTV-SegCo across the four date-blocked training–validation configurations is presented in **Figure 6** and **Table 8**. All models generated from these configurations were evaluated on the same fixed February 4 early-stage model-selection set. PTV-SegCo produced numerically higher mIoU, mAcc, and stem IoU values than PTV-Seg50 under each of the four configurations, with mean values of 89.05% ± 3.04%, 93.05% ± 2.66%, and 77.34% ± 6.17%, respectively. The corresponding values for PTV-Seg50 were 83.77% ± 0.51%, 88.87% ± 0.79%, and 66.64% ± 0.81%. The reported standard deviations reflect the sensitivity of models evaluated on the same model-selection set to changes in the composition of the training and validation dates. They do not represent variability across independent test folds. Furthermore, because repeated observations from the same cultivation trays occurred across the date-based subsets and the February 4 results contributed to architecture selection, these comparisons should be interpreted as internal model-development results rather than independent evidence of generalization or model stability on unseen cultivation trays.

**Table 8:** Performance comparison between PTV-Seg50 and PTV-SegCo across four date-blocked training–validation configurations.

Model	mIoU	mAcc	Stem IoU
PTV-Seg50	83.77 ± 0.51	88.87 ± 0.79	66.64 ± 0.81
PTV-SegCo	89.05 ± 3.04	93.05 ± 2.66	77.34 ± 6.17

Values are presented as mean ± standard deviation (%) across four date-blocked training–validation configurations. Models from all four configurations were evaluated on the same fixed February 4 early-stage model-selection set. Therefore, the reported standard deviations reflect sensitivity to the composition of the training and validation dates rather than variability across independent test folds.

It should be noted that, because the pot category accounts for a large proportion of the overall point cloud data, the allAcc metric is easily affected by the dominant category. Therefore, the allAcc of PTV-SegCo only increased from 99.552% to 99.610% compared with PTV-Seg50, representing an improvement of 0.058 percentage points. In contrast, mIoU and category-specific IoU metrics for stem and leaf can more accurately reflect the model’s ability to recognize plant organ structures and are therefore more suitable as the primary evaluation metrics for the task investigated in this study. Since the three point cloud categories (pot, stem, and leaf) exhibit obvious class imbalance, overall evaluation metrics alone cannot fully assess the model’s recognition capability for small-scale organ regions. Therefore, this study further calculated the class-wise Precision, Recall, and F1-score of PTV-SegCo by aggregating point-wise predictions from the fixed early-stage model-selection set, and the results are presented in **Table 9**.

**Table 9:** Class-wise precision, recall, and F1-score of PTV-SegCo on the fixed early-stage model-selection set.

Type	Precision (%)	Recall (%)	F1-score (%)
Pot	99.72	99.95	99.84
Stem	85.80	73.36	79.09
Leaf	93.13	89.54	91.30

Precision, Recall, and F1-score were calculated by aggregating point-wise predictions across the fixed February 4 early-stage model-selection samples. These samples were used during model development and therefore did not constitute an independent or untouched final test set. Class-wise IoU results are reported separately in the preceding model-evaluation analyses and are not included here because they were obtained using a different evaluation aggregation procedure.

Among the three semantic classes, the pot class showed the highest pooled point-wise classification metrics, with Precision, Recall, and F1-score all exceeding 99%. For the leaf category, Precision, Recall, and F1-score reached 93.13%, 89.54%, and 91.30%, respectively, indicating relatively high leaf-recognition performance on the fixed early-stage model-selection set. For the stem category, which was characterized by slender structures and a limited number of points, Precision, Recall, and F1-score were 85.80%, 73.36%, and 79.09%, respectively. The lower Recall than Precision for the stem category indicates that missed stem points were a more prominent source of error than false-positive stem predictions. This pattern is consistent with the structural characteristics of coriander seedlings, including the small proportion of stem points, limited spatial continuity of fine stems, ambiguous stem–leaf junctions, and local leaf occlusion.

**Figure 7:**
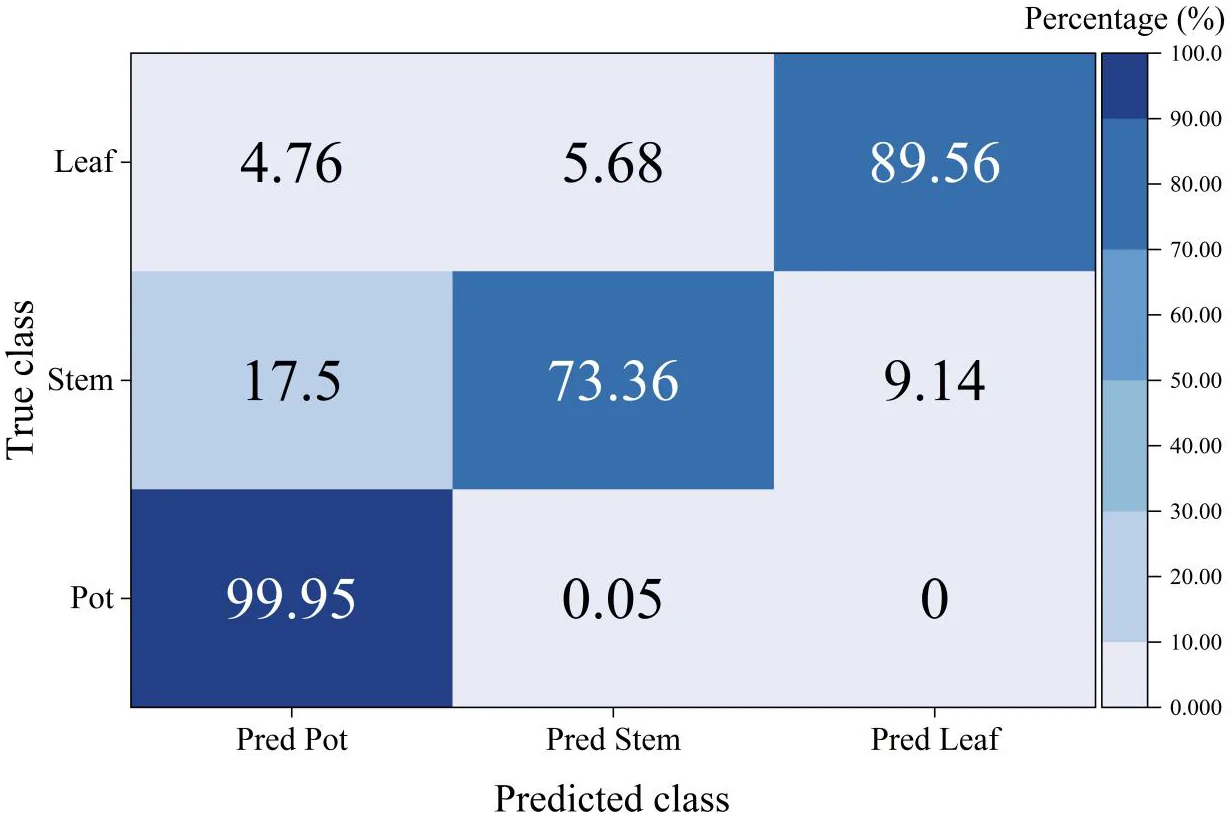
Heatmap of the confusion matrix.

The prediction relationships among different categories were further analyzed using a confusion matrix, as shown in **Figure 7**. The results indicate that the pot category achieved the most stable prediction performance, whereas a certain degree of confusion existed between the stem and leaf categories. This is related to the close structural relationship between stems and leaves in coriander seedlings, the similarity of color and spatial features in some regions, and occlusion effects. The confusion matrix results were consistent with the category-level evaluation metrics presented in **Table 9**, further indicating that the current limitations of the model are mainly concentrated in fine plant organ regions.

**Figure 8:**
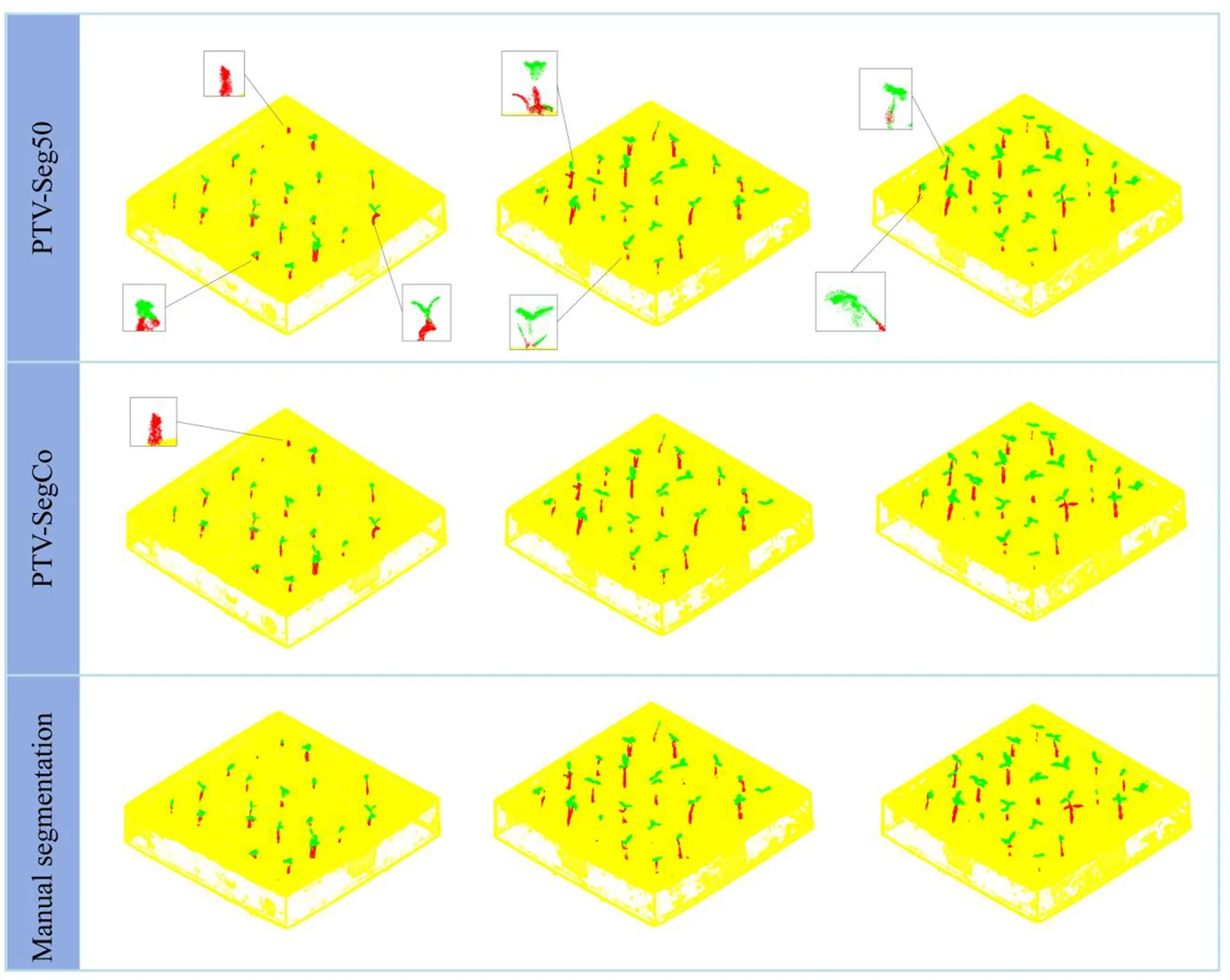
Qualitative visual analysis of coriander seedlings in the ablation experiments.

To intuitively evaluate the performance of the improved model in practical point cloud segmentation tasks, representative samples were further selected for visualization analysis in this study, as shown in **Figure 8**. The figure presents the manual annotation results, PTV-Seg50 prediction results, and PTV-SegCo prediction results. The results show that PTV-Seg50 can achieve basic semantic segmentation, but certain misclassification and missed segmentation problems remain in fine stem regions, stem–leaf junction regions, and overlapping leaf regions. In contrast, PTV-SegCo exhibited better performance in terms of overall structural integrity and boundary continuity, enabling more accurate discrimination between stem and leaf categories while reducing stem fragmentation and local category confusion. This improvement was mainly attributed to the synergistic effects of the three improved modules: the ECA module enhanced the representation capability of key structural features; the Gated Fusion module promoted the fusion of shallow spatial information and deep semantic information; and the CE-Dice joint loss function alleviated the class imbalance problem and improved the model’s learning capability for minority category regions.

In summary, under the original internal model-development protocol, the variants incorporating ECA, Gated Fusion, and CE-Dice Loss produced numerically higher segmentation metrics than the direct backbone PTV-Seg50 on the fixed early-stage model-selection set. Among the evaluated configurations, the complete PTV-SegCo configuration yielded the highest mAcc, mIoU, stem IoU, and leaf IoU values. These internal comparisons support the contribution of the three task-specific modifications relative to PTV-Seg50 under the stated protocol. However, because all configurations were evaluated on the same model-selection set and no additional model families were included, these results do not establish performance stability on independent test folds or superiority over other point-cloud segmentation architectures.

### 3.4 Three-dimensional structural phenotypic analysis of coriander seedlings under different NaCl treatments

#### 3.4.1 Individual-plant separation and quality-control results

The sensitivity of individual-plant separation to the DBSCAN neighborhood radius was evaluated using 20 representative scenes. Five candidate ϵ values ranging from 0.16 to 0.22 cm were examined while keeping MinPts fixed at 20. Across the tested range, the detected plant counts remained unchanged in 19 of the 20 scenes, indicating that the clustering results were generally insensitive to moderate variation in ϵ. The only count change occurred in sample 250_1_0208, for which 21 clusters were detected at ϵ = 0.16 and 0.18 cm, whereas 20 clusters were obtained at ϵ = 0.19, 0.20, and 0.22 cm (**Supplementary Table 2**).

Visual inspection of the pseudo-colored clustering results showed that the additional cluster detected at the smaller ϵ values in sample 250_1_0208 represented a disconnected fragment of the same biological plant rather than an independent seedling. Increasing ϵ to 0.19 cm connected these fragments into a single plant instance without visible merging of neighboring seedlings. Based on the parameter-sensitivity results and visual inspection, ϵ = 0.19 cm with MinPts = 20 was therefore adopted for the final batch individual-plant separation.

Using the selected DBSCAN parameters, the initial separation of all 60 scenes yielded 1,145 candidate plant clusters. Subsequent manual quality control identified seven cases in which disconnected clusters represented fragments of the same biological plant and one false cluster corresponding to an impurity/noise component. Seven merge operations and one cluster removal were therefore applied across six scenes. After these corrections, 1,137 QC-valid individual-plant observations were retained for subsequent structural phenotypic analysis. No manually confirmed under-segmentation requiring the separation of multiple biological plants from a single DBSCAN cluster was identified during the final quality-control procedure. The complete manually confirmed correction records are provided in **Supplementary Table 3**.

The predefined 5 × 5 sowing layout was used only as an auxiliary spatial reference during quality control and was not used to enforce one-to-one longitudinal correspondence of individual seedlings across acquisition dates. Because the final statistical analysis did not rely on cross-date individual-plant identities, each acquisition scene was quality-controlled independently, and the QC-valid individual-plant measurements were subsequently summarized at the cultivation-tray-by-acquisition-time level.

#### 3.4.2 Structural phenotypic responses to NaCl treatments

**Figure 9:**
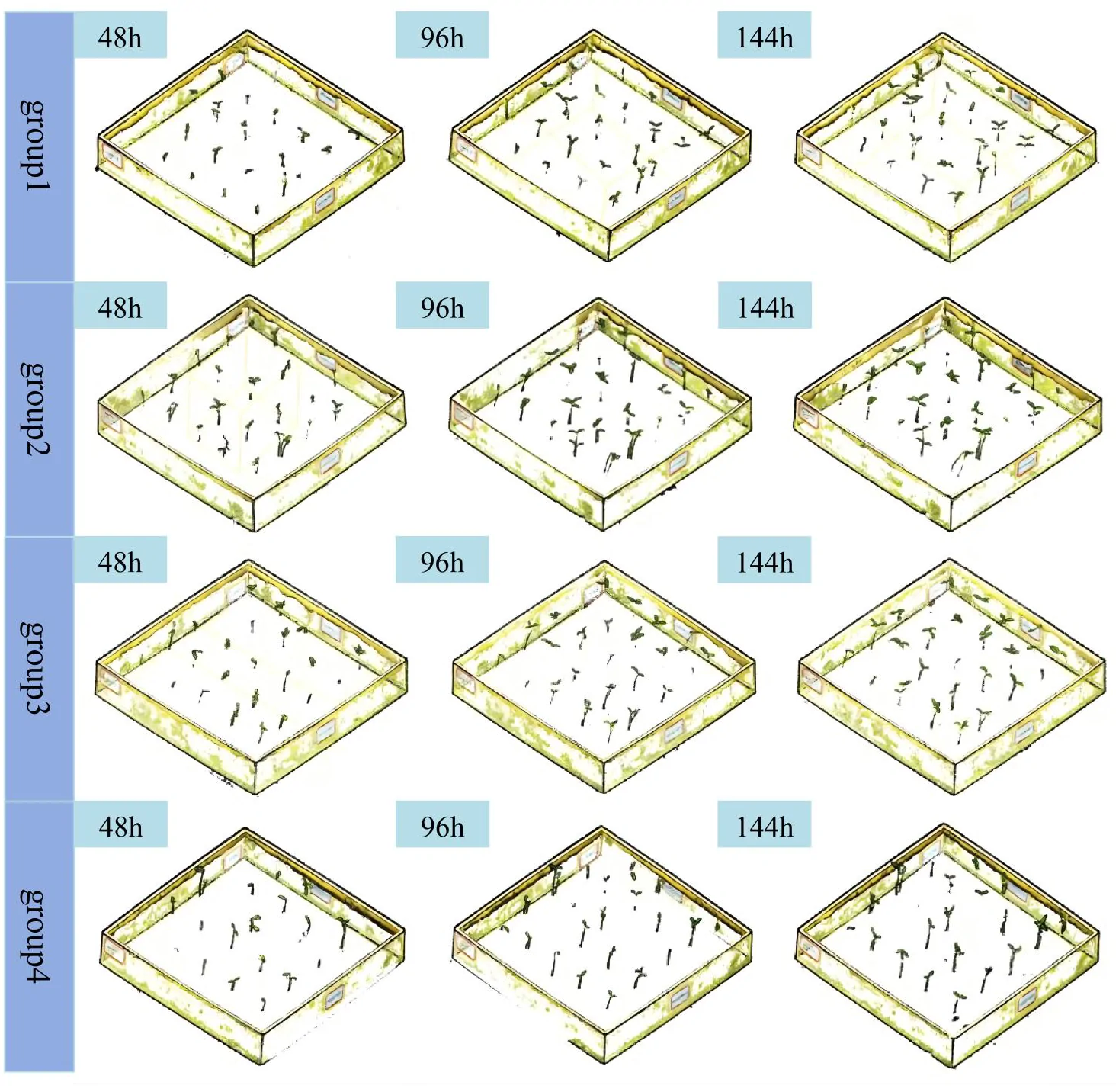
3D point cloud reconstruction images of coriander seedlings during growth.

**Figure 10:**
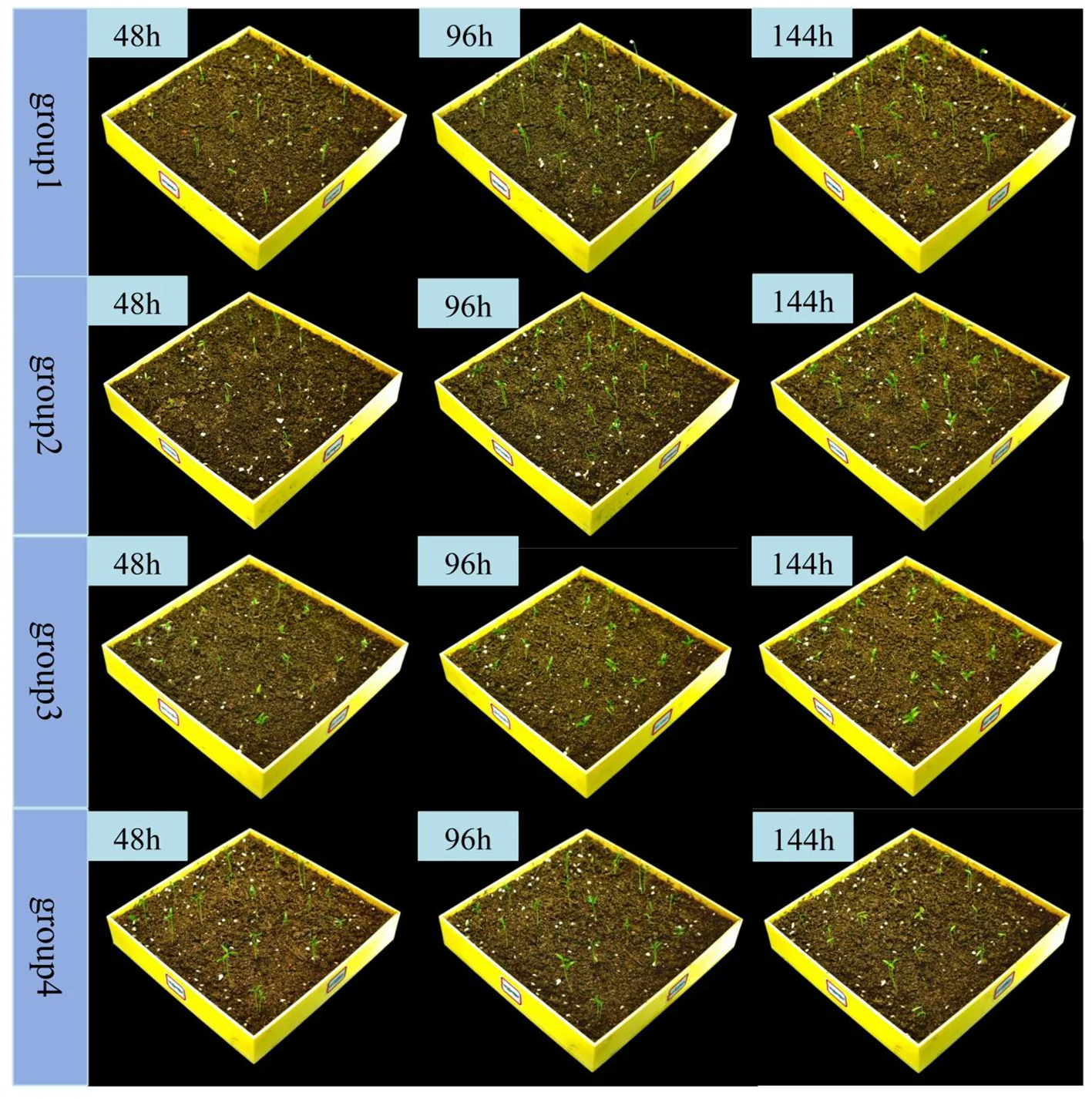
Axonometric images of coriander seedlings during growth.

Based on the metric-calibrated and quality-controlled individual-plant point clouds described in Section 2.4, four structural phenotypic descriptors, including plant height, projected area, voxel occupancy volume, and leaf point ratio, were obtained to characterize the structural responses of coriander seedlings under different NaCl treatments. Six NaCl concentrations were evaluated, including 0 (CK), 50, 100, 150, 200, and 250 mmol L^−1^, and observations were conducted at 48, 72, 96, 120, and 144 h. Representative three-dimensional point-cloud reconstructions under the different NaCl treatments are shown in **Figure 9**, while the corresponding axonometric images are presented in **Figure 10**. Together, these images provide a qualitative representation of the temporal changes in seedling spatial structure during the observation period.

**Figure 11:**
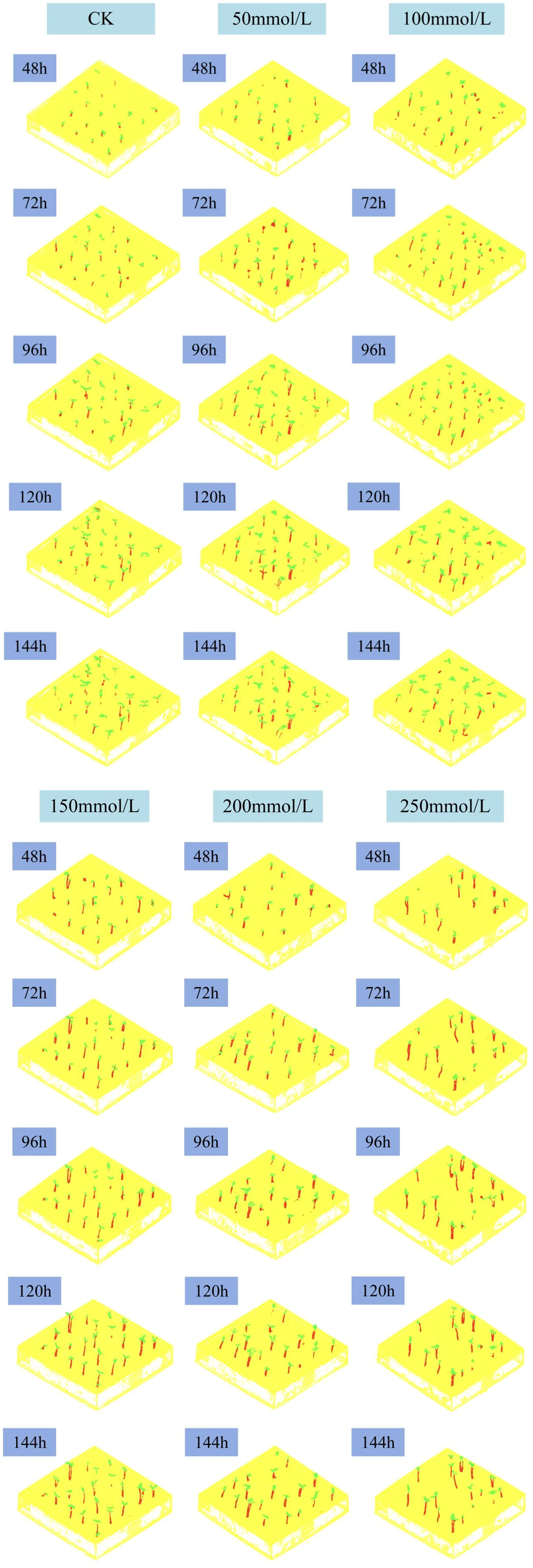
Semantic segmentation of coriander seedlings Using PTV-SegCo.

**Figure 12:**
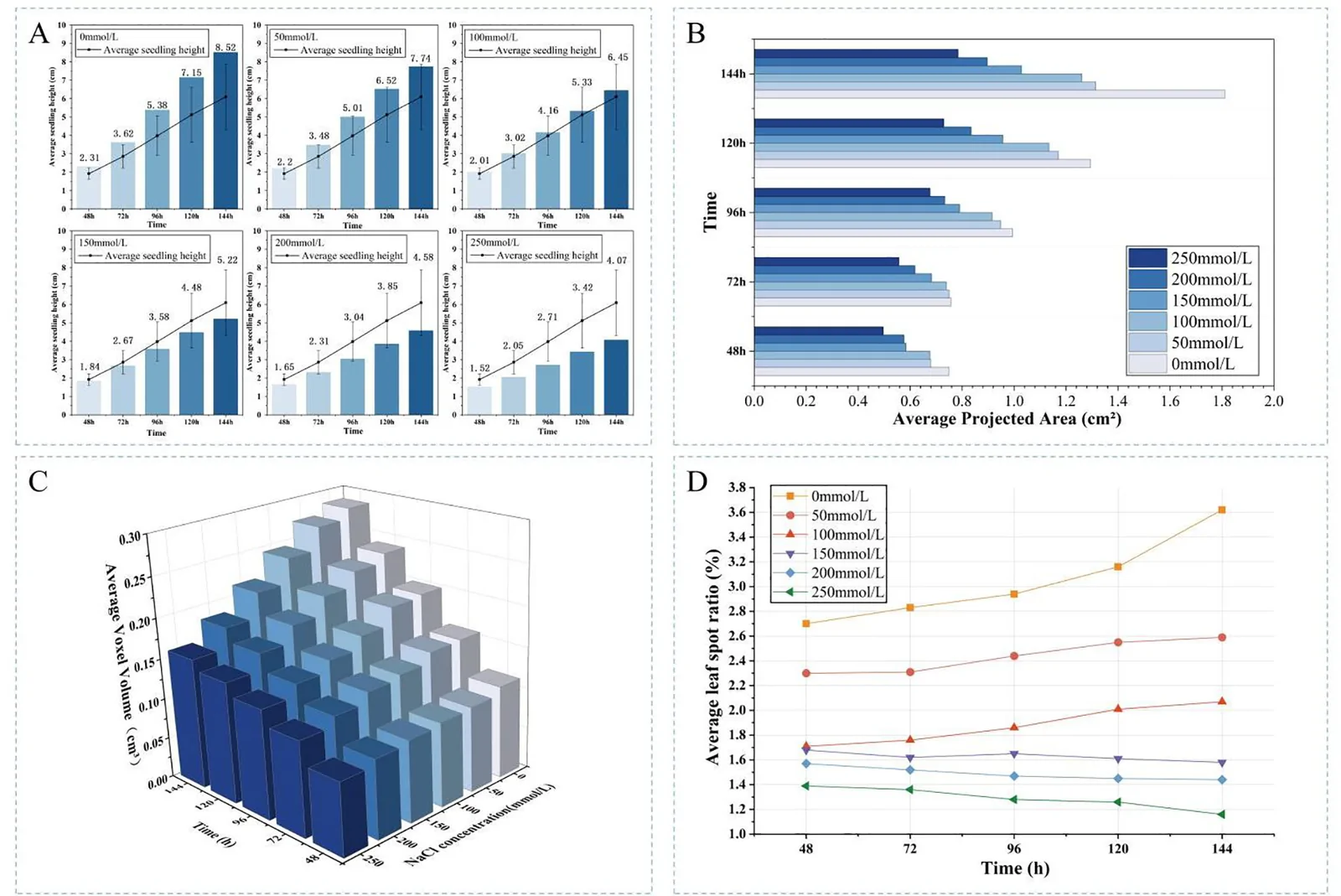
Temporal variation of structural phenotypic descriptors of coriander seedlings under different NaCl treatments from 48 to 144 h. **(A)** Average plant height; **(B)** average projected area; **(C)** average voxel occupancy volume; and **(D)** average leaf point ratio. Six NaCl concentrations were evaluated: 0 (CK), 50, 100, 150, 200, and 250 mmol L^−1^. Plant height, projected area, and voxel occupancy volume are presented in cm, cm^2^, and cm^3^, respectively, whereas leaf point ratio is expressed as a percentage (%).

Before statistical interpretation of the structural phenotypic descriptors, the assumptions of the tray-level linear mixed-effects models described in Section 2.5 were examined. As shown in **Figures 5A–C**, the standardized residuals for plant height, natural-log-transformed projected area, and voxel occupancy volume generally followed the theoretical normal reference line and remained largely within the 95% reference envelopes, indicating no substantial departure from the residual normality assumption for these dimensional descriptors. Projected area is presented on its original cm^2^ scale in the descriptive analyses below, whereas its natural-log-transformed values were used for mixed-effects modeling as described in Section 2.5. Representative organ-level semantic segmentation results produced by PTV-SegCo are shown in **Figure 11**, illustrating the spatial distributions of the pot, stem, and leaf categories under the six NaCl treatments across the observation period. Based on these segmentation results, the temporal variation of plant height, projected area, voxel occupancy volume, and leaf point ratio was quantified and is summarized in **Figure 12**.

Plant height was used to characterize the vertical structural development of coriander seedlings. As shown in **Figure 12A**, plant height increased continuously from 48 to 144 h in all treatment groups, whereas the magnitude of increase progressively decreased with increasing NaCl concentration. The CK group showed the greatest increase, from 2.31 cm at 48 h to 8.52 cm at 144 h. At the final observation time, plant heights under the 50, 100, 150, 200, and 250 mmol L^−1^ treatments were 7.74, 6.45, 5.22, 4.58, and 4.07 cm, respectively. Compared with the CK group, these values were lower by 9.15%, 24.30%, 38.73%, 46.24%, and 52.23%, respectively. Differences among NaCl treatments became increasingly apparent with seedling development, particularly from 96 to 144 h. The 0 and 50 mmol L^−1^ groups maintained relatively greater vertical development, whereas the 150–250 mmol L^−1^ treatments showed progressively smaller plant heights. Thus, the vertical extent of the reconstructed seedlings exhibited an increasingly distinct concentration-related gradient over time. The tray-level LMM indicated no significant overall main effect of NaCl concentration on plant height (Wald χ^2^(5) = 2.36, p = 0.797), whereas the effect of acquisition time was significant (Wald χ^2^(4) = 19.13, p< 0.001). A significant NaCl concentration × acquisition time interaction was also detected (Wald χ^2^(20) = 42.03, p = 0.0027), indicating that the temporal pattern of plant height differed among NaCl treatments.

Projected area was used to characterize the horizontal spatial coverage of the reconstructed coriander seedlings. As shown in **Figure 12B**, projected area generally increased with acquisition time in all treatment groups, but the extent of horizontal expansion decreased as NaCl concentration increased. In the CK group, projected area increased from 0.749 cm^2^ at 48 h to 1.811 cm^2^ at 144 h. At 144 h, the corresponding projected areas under the 50, 100, 150, 200, and 250 mmol L^−1^ treatments were 1.314, 1.260, 1.028, 0.896, and 0.784 cm^2^, respectively, representing reductions of 27.44%, 30.43%, 43.24%, 50.52%, and 56.71% relative to the CK group. At the early observation times, the differences among several treatment groups were relatively limited, whereas clearer separation emerged during later development. In particular, projected area continued to increase markedly in the CK group from 96 to 144 h, while the increase was progressively restricted in the higher-concentration NaCl groups. These results indicate increasingly differentiated horizontal spatial expansion of reconstructed seedling structures with increasing NaCl concentration. For natural-log-transformed projected area, the overall main effect of NaCl concentration was not significant (Wald χ^2^(5) = 6.06, p = 0.300), whereas acquisition time showed a significant effect (Wald χ^2^(4) = 102.45, p< 0.001). The NaCl concentration × acquisition time interaction was also significant (Wald χ^2^(20) = 46.63, p< 0.001), indicating that differences in projected area among NaCl treatments changed over the observation period.

Voxel occupancy volume was used to describe the three-dimensional spatial occupancy of the reconstructed seedling point clouds rather than the true physical volume or biomass of plant tissues. As shown in **Figure 12C**, voxel occupancy volume increased over time in all six treatment groups, while progressively lower values were observed with increasing NaCl concentration. The CK group increased from 0.113 cm^3^ at 48 h to 0.286 cm^3^ at 144 h. At 144 h, voxel occupancy volumes under the 50, 100, 150, 200, and 250 mmol L^−1^ treatments were 0.273, 0.245, 0.215, 0.185, and 0.160 cm^3^, respectively. Relative to the CK group, these values were reduced by 4.55%, 14.34%, 24.83%, 35.31%, and 44.06%, respectively. The difference between the CK and 50 mmol L^−1^ groups remained comparatively small throughout the observation period, whereas increasingly clear reductions were observed at concentrations of 100 mmol L^−1^ and above. The progressive divergence of the treatment groups from 96 to 144 h indicates that the three-dimensional occupancy of the reconstructed seedling point clouds became increasingly differentiated among NaCl concentrations. For voxel occupancy volume, the overall main effect of NaCl concentration was not significant (Wald χ^2^(5) = 2.90, p = 0.715), whereas acquisition time had a significant effect (Wald χ^2^(4) = 13.09, p = 0.0108). A significant NaCl concentration × acquisition time interaction was observed (Wald χ^2^(20) = 45.83, p< 0.001), indicating treatment-dependent differences in the temporal development of three-dimensional point-cloud occupancy.

The leaf point ratio was expressed as a percentage-based structural descriptor derived from the segmented point-cloud data. As shown in **Figure 12D**, its temporal pattern differed from those of plant height, projected area, and voxel occupancy volume. In the CK group, the leaf point ratio increased from 2.70% at 48 h to 3.62% at 144 h. The 50 and 100 mmol L^−1^ groups also showed an overall increase during the observation period, reaching 2.59% and 2.07%, respectively, at 144 h. By contrast, the higher-concentration groups showed relatively stable or decreasing trajectories. The 150 mmol L^−1^ group varied only slightly from 1.68% at 48 h to 1.58% at 144 h, whereas the 200 and 250 mmol L^−1^ groups decreased from 1.57% and 1.39% to 1.44% and 1.16%, respectively. At 144 h, the values under the 50, 100, 150, 200, and 250 mmol L^−1^ treatments were 28.45%, 42.82%, 56.35%, 60.22%, and 67.96% lower than that of the CK group, respectively. Thus, while the CK and lower-concentration treatments exhibited increasing values over time, this temporal increase became weaker and ultimately reversed under the higher NaCl concentrations. Because this descriptor is derived from the relative distribution of segmented point-cloud categories, it should be interpreted as a reconstruction-based structural characteristic rather than as a direct measure of leaf biomass, actual leaf area, or biomass allocation.

Overall, **Figures 9**-**12** provide complementary qualitative and quantitative characterization of the structural development of coriander seedlings under different NaCl treatments. The reconstructed three-dimensional point clouds in **Figure 9** and the corresponding axonometric images in **Figure 10** illustrate the temporal morphological changes observed during seedling development. **Figure 11** presents the organ-level semantic segmentation results that formed the basis for subsequent individual-plant separation and structural descriptor extraction, while **Figure 12** summarizes the temporal variation in plant height, projected area, voxel occupancy volume, and leaf point ratio under the six NaCl treatments. Across the three dimensional structural descriptors, increasing NaCl concentration was generally associated with progressively smaller plant height, projected area, and voxel occupancy volume, and the separation among treatment groups became more pronounced at later acquisition times. In contrast, leaf point ratio showed a different temporal pattern, increasing in the CK and lower-concentration groups while remaining relatively stable or decreasing under the higher NaCl treatments. Together, these results demonstrate concentration-dependent differences in the reconstructed three-dimensional structural development of coriander seedlings during the observation period. The extracted variables are interpreted as structural phenotypic descriptors, and the observed trends are not used to infer specific physiological mechanisms.

#### 3.4.3 Independent validation of plant height and projected area

**Figure 13:**
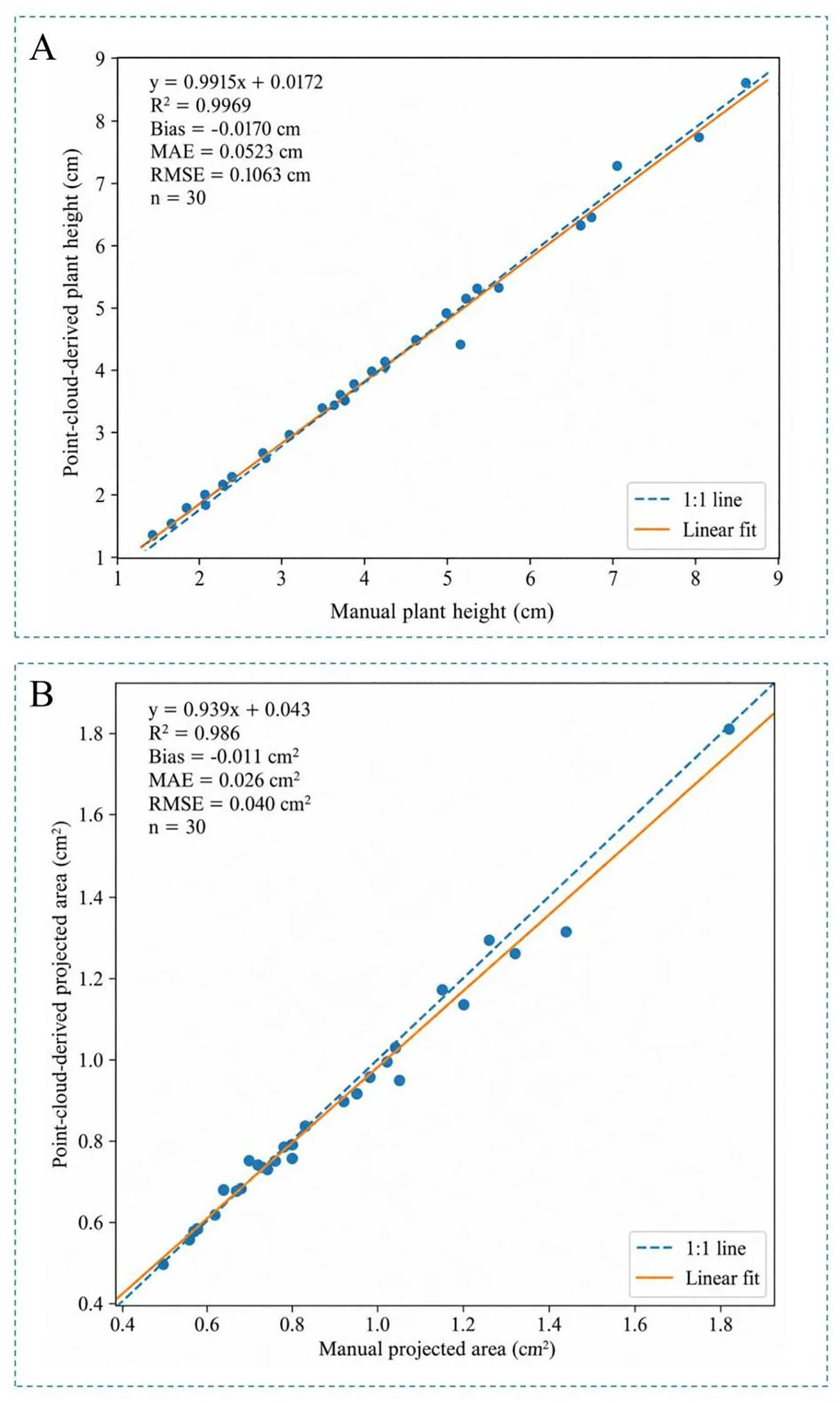
Independent validation of reconstruction-derived structural phenotypic descriptors against manual reference measurements. **(A)** Comparison between manual and point-cloud-derived plant height; **(B)** comparison between manual and point-cloud-derived projected area. Each point represents one paired treatment-by-time observation (n = 30; six NaCl concentrations × five acquisition times). The blue dashed line represents the 1:1 reference line, and the orange solid line represents the fitted linear regression. Bias was calculated as the point-cloud-derived value minus the manual reference value. R2, MAE, and RMSE denote the coefficient of determination, mean absolute error, and root mean square error, respectively.

To evaluate the measurement accuracy of the reconstruction-derived structural descriptors, plant height and projected area were independently compared with the corresponding manual reference measurements across 30 paired treatment-by-time observations, covering six NaCl concentrations and five acquisition times. As shown in **Figure 13**, the point-cloud-derived measurements were closely distributed around the 1:1 reference line for both traits.

For plant height, linear regression between the manual and point-cloud-derived measurements yielded y=0.9915x+0.0172, with R2 = 0.9969 (**Figure 13A**). The mean bias was −0.0170 cm, while the MAE and RMSE were 0.0523 and 0.1063 cm, respectively. The regression slope was close to 1 and the bias was close to zero, indicating close agreement between the two measurement approaches over the evaluated range.

Similarly, the point-cloud-derived projected area showed strong agreement with the manual reference measurements, with a regression equation of y=0.939x+0.043 and R2 = 0.986 (**Figure 13B**). The mean bias was −0.011 cm^2^, and the MAE and RMSE were 0.026 and 0.040 cm^2^, respectively. The slightly negative bias and regression slope below 1 indicated a small tendency toward underestimation, particularly at larger projected-area values; however, the overall magnitude of the measurement error remained low.

Taken together, these results provide independent quantitative support for the measurement accuracy of the reconstruction-derived plant height and projected area under the conditions investigated in this study. Voxel occupancy volume and leaf point ratio were not included in this external validation because they are reconstruction-specific structural descriptors without directly equivalent conventional reference measurements; therefore, they continue to be interpreted as point-cloud-derived structural descriptors rather than directly validated physical or physiological traits.

## 4 Discussion

This study addressed several challenges in three-dimensional point cloud structural analysis of coriander seedlings under salt stress conditions, including leaf overlap, small stem scale, unclear boundaries in stem–leaf junction regions, and imbalanced category distributions. Based on the PTV-Seg50 framework, an optimized model, PTV-SegCo, was developed, and a three-dimensional structural phenotyping analysis pipeline based on semantic segmentation results was further established. Compared with traditional manual measurement methods, the proposed approach enables automated acquisition of plant spatial structural information using three-dimensional point cloud data, providing a feasible technical pathway for continuous and non-destructive structural monitoring of small plant seedlings under salt stress conditions (Fahlgren et al., 2015; Gill et al., 2022).

From the perspective of model optimization, the performance improvement of PTV-SegCo mainly resulted from feature enhancement, feature fusion, and loss function optimization strategies designed according to the structural characteristics of coriander seedlings. The ECA module improved the feature representation capability of the network for fine organ structures and critical local regions by adaptively adjusting feature channel weights. The gated shallow–deep feature fusion module enhanced the collaborative utilization of spatial detail information and high-level semantic information, contributing to improved recognition of fine stems and complex stem–leaf junction regions. The Cross-Entropy–Dice joint loss function alleviated the training bias caused by differences in point numbers among categories and improved the model’s learning capability for minority category regions. Therefore, this study did not propose an entirely new theoretical framework for point cloud segmentation; rather, it adapted and integrated existing deep learning optimization strategies according to specific plant structural characteristics to meet the requirements of organ-level point cloud segmentation for coriander seedlings. Accordingly, the comparison with PTV-Seg50 should be interpreted as a controlled backbone-level analysis of the proposed modifications rather than as a comprehensive assessment of competitiveness across different segmentation architectures.

In addition to the internal comparison of point cloud semantic segmentation models, another contribution of this study is the establishment of a semantic segmentation-based data foundation for structural descriptor analysis of coriander seedlings under salt stress conditions. The structural parameters extracted from accurate segmentation results, including plant height, projected area, voxel occupancy volume, and leaf point ratio, can describe seedling structural changes at different spatial scales; the definition and interpretation of these indicators are summarized in **Table 10**. Among these parameters, plant height and projected area reflect vertical and horizontal spatial expansion capacities, respectively; voxel occupancy volume provides an integrated description of three-dimensional spatial occupancy characteristics; and leaf point ratio assists in analyzing changes in the relative distribution of different organ categories within the point cloud structure. Compared with single two-dimensional image information, three-dimensional point clouds preserve spatial topological relationships of plants and are therefore more suitable for analyzing structural changes under conditions involving leaf overlap and organ interlacing.

**Table 10:** Definition and interpretation of extracted structural phenotypic indicators.

Indicator	Definition	Biological interpretation limitation
Plant height	Z-axis range	Structural descriptor
Projected area	2D projection area	Affected by viewing geometry
Voxel occupancy volume	Occupied voxels	Not true biomass
Leaf point ratio	Leaf point proportion	Affected by reconstruction density

It should be emphasized that the three-dimensional structural phenotypic descriptors extracted in this study were primarily derived from point-cloud reconstruction and semantic segmentation results and were used to characterize spatial structural changes of coriander seedlings. Plant height and projected area were independently evaluated against manual reference measurements in the present study. The high agreement observed for plant height (R2 = 0.9969, MAE = 0.0523 cm, RMSE = 0.1063 cm) and projected area (R2 = 0.986, MAE = 0.026 cm^2^, RMSE = 0.040 cm^2^) provides quantitative support for the measurement accuracy of these two reconstruction-derived descriptors under the present experimental conditions. Nevertheless, voxel occupancy volume and leaf point ratio remain reconstruction-specific descriptors and were not independently validated against direct physical or physiological reference measurements. Therefore, these parameters should not be directly equated with biomass, actual tissue volume, biomass allocation, or physiological processes. Future studies should further validate reconstruction-derived descriptors using destructive measurements, direct leaf-area determination, biomass analysis, and physiological and biochemical indicators under broader experimental conditions.

Meanwhile, this study still has several limitations. First, the original acquisition-date-based protocol did not ensure independence at the cultivation-tray level because repeated observations of the same trays occurred across different subsets. Moreover, because the February 4 samples were used for ablation comparison and final architecture selection, the study did not include a strictly untouched final test set. Therefore, the reported segmentation metrics should be interpreted as internal comparative results under the present protocol rather than as an unbiased estimate of generalization to unseen cultivation trays. Future studies should retain the complete time series of each tray within a single partition and reserve unseen trays for final evaluation. Second, PTV-SegCo was compared only with its direct backbone, PTV-Seg50. This controlled comparison was designed to evaluate the effects of the proposed task-specific modifications within the same backbone framework rather than to establish superiority over different point-cloud segmentation model families. Classical point-based methods, graph- or convolution-based architectures, recent transformer-based models, and plant-specific segmentation methods were not systematically retrained and evaluated under the same cultivation-tray-grouped data partitions. Consequently, the current results do not establish the broad competitiveness of PTV-SegCo against state-of-the-art point-cloud segmentation architectures. Future studies should conduct unified benchmarking using cultivation-tray-independent partitions, larger annotated datasets, and identical preprocessing, optimization, model-selection, and evaluation settings. Third, the dataset size in this study was relatively limited, containing only 60 three-dimensional point cloud samples of coriander seedlings, and the experimental materials were derived from a single variety and a single cultivation environment. Therefore, the applicability of the PTV-SegCo model and the salt stress response patterns obtained from three-dimensional structural parameters under different varieties, environmental conditions, and practical production scenarios requires further validation. Future research should expand sample sizes and introduce more plant types and complex environmental conditions to improve model universality. Furthermore, due to the natural overlap and unclear boundaries among plant organs, this study did not conduct an inter-annotator consistency evaluation. Future work will evaluate annotation reliability through independent labeling by multiple experts and consistency analysis based on larger-scale datasets. In addition, the effects of factors such as voxel size, point cloud density, and different data augmentation strategies on the segmentation performance of fine organs require more systematic sensitivity analyses.

Overall, this study established a complete analytical workflow from three-dimensional point cloud acquisition, semantic segmentation, to structural phenotypic trait extraction, providing an automated and non-destructive approach for investigating spatial structural changes of coriander seedlings under salt stress conditions. In the future, integrating multi-scale phenotypic data, physiological and biochemical measurements, and larger-scale cross-environment datasets may further enhance the application value of three-dimensional plant phenotyping approaches in salt stress response evaluation and salt-tolerant material screening.

## Data Availability

The original contributions presented in the study are included in the article/**Supplementary Material**. Further inquiries can be directed to the corresponding author.

## Supplementary material

Supplementary Table 1 Sample-level point distribution and class proportions of the annotated coriander seedling point-cloud dataset.

Supplementary Table 2 Sensitivity analysis of the DBSCAN neighborhood radius for individual-plant separation.

Supplementary Table 3 Manual quality-control corrections applied after DBSCAN-based individual-plant separation.
