# Supplementary material for: Three-dimensional organ segmentation and structural phenotyping of salt-stressed coriander seedlings using the optimized point transformer-based model PTV-SegCo
Source: Front Plant Sci. 2026 Aug 31;17:1883203. doi: 10.3389/fpls.2026.1883203 (PMC13572714; doi:10.3389/fpls.2026.1883203)
Supplement: Supplementary file 1 [file Table1.docx]

Table S1. Sample-level point distribution and class proportions of the annotated coriander seedling point-cloud dataset

| **NaCl (mmol/L)** | **Date** | **Tray** | **Sample_ID** | **Detected_plants** | **Valid_plants** | **Total_points** | **Pot_points** | **Stem_points** | **Leaf_points** | **Pot_ratio(%)** | **Stem_ratio(%)** | **Leaf_ratio(%)** |
| --- | --- | --- | --- | --- | --- | --- | --- | --- | --- | --- | --- | --- |
| 0 | 02/04 | 1 | 0_1_0204 | 19 | 18 | 1177972 | 1132470 | 12754 | 32748 | 96.14 | 1.08 | 2.78 |
| 0 | 02/05 | 1 | 0_1_0205 | 20 | 20 | 1127438 | 1085256 | 7231 | 34951 | 96.26 | 0.64 | 3.10 |
| 0 | 02/06 | 1 | 0_1_0206 | 19 | 18 | 1177967 | 1132112 | 7453 | 38402 | 96.11 | 0.63 | 3.26 |
| 0 | 02/07 | 1 | 0_1_0207 | 19 | 19 | 1166165 | 1116899 | 11016 | 38250 | 95.78 | 0.94 | 3.28 |
| 0 | 02/08 | 1 | 0_1_0208 | 19 | 19 | 1184201 | 1127478 | 9947 | 46776 | 95.21 | 0.84 | 3.95 |
| 0 | 02/04 | 2 | 0_2_0204 | 19 | 19 | 1158696 | 1112876 | 15462 | 30358 | 96.05 | 1.33 | 2.62 |
| 0 | 02/05 | 2 | 0_2_0205 | 17 | 17 | 1093215 | 1046773 | 18456 | 27986 | 95.75 | 1.69 | 2.56 |
| 0 | 02/06 | 2 | 0_2_0206 | 19 | 19 | 1169847 | 1119395 | 19802 | 30650 | 95.69 | 1.69 | 2.62 |
| 0 | 02/07 | 2 | 0_2_0207 | 21 | 21 | 1110310 | 1067785 | 8772 | 33753 | 96.17 | 0.79 | 3.04 |
| 0 | 02/08 | 2 | 0_2_0208 | 20 | 20 | 1113028 | 1070969 | 5441 | 36618 | 96.22 | 0.49 | 3.29 |
| 50 | 02/04 | 1 | 50_1_0204 | 19 | 19 | 1128755 | 1092697 | 5356 | 30702 | 96.81 | 0.47 | 2.72 |
| 50 | 02/05 | 1 | 50_1_0205 | 22 | 22 | 1146373 | 1101474 | 17386 | 27513 | 96.08 | 1.52 | 2.40 |
| 50 | 02/06 | 1 | 50_1_0206 | 22 | 22 | 1135555 | 1095230 | 11709 | 28616 | 96.45 | 1.03 | 2.52 |
| 50 | 02/07 | 1 | 50_1_0207 | 25 | 25 | 1149574 | 1105102 | 14009 | 30463 | 96.13 | 1.22 | 2.65 |
| 50 | 02/08 | 1 | 50_1_0208 | 24 | 24 | 1142095 | 1101764 | 9495 | 30836 | 96.47 | 0.83 | 2.70 |
| 50 | 02/04 | 2 | 50_2_0204 | 12 | 12 | 1144047 | 1116841 | 5698 | 21508 | 97.62 | 0.50 | 1.88 |
| 50 | 02/05 | 2 | 50_2_0205 | 21 | 21 | 1182109 | 1144876 | 10990 | 26243 | 96.85 | 0.93 | 2.22 |
| 50 | 02/06 | 2 | 50_2_0206 | 22 | 22 | 1176108 | 1135067 | 13285 | 27756 | 96.51 | 1.13 | 2.36 |
| 50 | 02/07 | 2 | 50_2_0207 | 23 | 23 | 1186449 | 1142342 | 15039 | 29068 | 96.28 | 1.27 | 2.45 |
| 50 | 02/08 | 2 | 50_2_0208 | 23 | 23 | 1164137 | 1124230 | 11037 | 28870 | 96.57 | 0.95 | 2.48 |
| 100 | 02/04 | 1 | 100_1_0204 | 20 | 20 | 1157325 | 1126789 | 8200 | 22336 | 97.36 | 0.71 | 1.93 |
| 100 | 02/05 | 1 | 100_1_0205 | 23 | 23 | 1159306 | 1125631 | 12344 | 21331 | 97.10 | 1.06 | 1.84 |
| 100 | 02/06 | 1 | 100_1_0206 | 23 | 23 | 1159306 | 1124187 | 12860 | 22259 | 96.97 | 1.11 | 1.92 |
| 100 | 02/07 | 1 | 100_1_0207 | 22 | 22 | 1155902 | 1119818 | 11810 | 24274 | 96.88 | 1.02 | 2.10 |
| 100 | 02/08 | 1 | 100_1_0208 | 22 | 22 | 1132361 | 1100520 | 7495 | 24346 | 97.19 | 0.66 | 2.15 |
| 100 | 02/04 | 2 | 100_2_0204 | 13 | 13 | 1173898 | 1152030 | 4377 | 17491 | 98.14 | 0.37 | 1.49 |
| 100 | 02/05 | 2 | 100_2_0205 | 15 | 15 | 1185317 | 1155295 | 10109 | 19913 | 97.47 | 0.85 | 1.68 |
| 100 | 02/06 | 2 | 100_2_0206 | 15 | 15 | 1181451 | 1148899 | 11286 | 21266 | 97.24 | 0.96 | 1.80 |
| 100 | 02/07 | 2 | 100_2_0207 | 16 | 16 | 1179181 | 1143961 | 12580 | 22640 | 97.01 | 1.07 | 1.92 |
| 100 | 02/08 | 2 | 100_2_0208 | 17 | 17 | 1176421 | 1145938 | 7072 | 23411 | 97.41 | 0.60 | 1.99 |
| 150 | 02/04 | 1 | 150_1_0204 | 13 | 13 | 1144643 | 1111502 | 13110 | 20031 | 97.10 | 1.15 | 1.75 |
| 150 | 02/05 | 1 | 150_1_0205 | 15 | 15 | 1126559 | 1088324 | 19083 | 19152 | 96.61 | 1.69 | 1.70 |
| 150 | 02/06 | 1 | 150_1_0206 | 17 | 17 | 1153235 | 1110075 | 23324 | 19836 | 96.26 | 2.02 | 1.72 |
| 150 | 02/07 | 1 | 150_1_0207 | 17 | 17 | 1137392 | 1093833 | 24451 | 19108 | 96.17 | 2.15 | 1.68 |
| 150 | 02/08 | 1 | 150_1_0208 | 15 | 15 | 1126600 | 1083512 | 24499 | 18589 | 96.18 | 2.17 | 1.65 |
| 150 | 02/04 | 2 | 150_2_0204 | 17 | 17 | 1181852 | 1143770 | 19054 | 19028 | 96.78 | 1.61 | 1.61 |
| 150 | 02/05 | 2 | 150_2_0205 | 18 | 18 | 1206289 | 1165634 | 22078 | 18577 | 96.63 | 1.83 | 1.54 |
| 150 | 02/06 | 2 | 150_2_0206 | 19 | 19 | 1189804 | 1151025 | 19980 | 18799 | 96.74 | 1.68 | 1.58 |
| 150 | 02/07 | 2 | 150_2_0207 | 19 | 19 | 1181961 | 1140293 | 23466 | 18202 | 96.47 | 1.99 | 1.54 |
| 150 | 02/08 | 2 | 150_2_0208 | 19 | 19 | 1158559 | 1123574 | 17491 | 17494 | 96.98 | 1.51 | 1.51 |
| 200 | 02/04 | 1 | 200_1_0204 | 14 | 14 | 1165259 | 1134836 | 11546 | 18877 | 97.39 | 0.99 | 1.62 |
| 200 | 02/05 | 1 | 200_1_0205 | 16 | 15 | 1147504 | 1114798 | 14805 | 17901 | 97.15 | 1.29 | 1.56 |
| 200 | 02/06 | 1 | 200_1_0206 | 19 | 19 | 1174638 | 1139756 | 17028 | 17854 | 97.03 | 1.45 | 1.52 |
| 200 | 02/07 | 1 | 200_1_0207 | 17 | 17 | 1160981 | 1127903 | 15779 | 17299 | 97.15 | 1.36 | 1.49 |
| 200 | 02/08 | 1 | 200_1_0208 | 16 | 16 | 1151201 | 1117667 | 16496 | 17038 | 97.09 | 1.43 | 1.48 |
| 200 | 02/04 | 2 | 200_2_0204 | 22 | 22 | 1071560 | 1047456 | 7816 | 16288 | 97.75 | 0.73 | 1.52 |
| 200 | 02/05 | 2 | 200_2_0205 | 22 | 22 | 1062362 | 1040436 | 6203 | 15723 | 97.94 | 0.58 | 1.48 |
| 200 | 02/06 | 2 | 200_2_0206 | 25 | 25 | 1099650 | 1071857 | 12178 | 15615 | 97.47 | 1.11 | 1.42 |
| 200 | 02/07 | 2 | 200_2_0207 | 25 | 25 | 1067280 | 1039627 | 12604 | 15049 | 97.41 | 1.18 | 1.41 |
| 200 | 02/08 | 2 | 200_2_0208 | 24 | 24 | 1043683 | 1015511 | 13560 | 14612 | 97.30 | 1.30 | 1.40 |
| 250 | 02/04 | 1 | 250_1_0204 | 19 | 16 | 384986 | 365391 | 14090 | 5505 | 94.91 | 3.66 | 1.43 |
| 250 | 02/05 | 1 | 250_1_0205 | 17 | 17 | 400673 | 370889 | 24175 | 5609 | 92.57 | 6.03 | 1.40 |
| 250 | 02/06 | 1 | 250_1_0206 | 23 | 23 | 1109864 | 1077893 | 17321 | 14650 | 97.12 | 1.56 | 1.32 |
| 250 | 02/07 | 1 | 250_1_0207 | 22 | 21 | 1108525 | 1075620 | 18605 | 14300 | 97.03 | 1.68 | 1.29 |
| 250 | 02/08 | 1 | 250_1_0208 | 20 | 19 | 1086107 | 1053185 | 19997 | 12925 | 96.97 | 1.84 | 1.19 |
| 250 | 02/04 | 2 | 250_2_0204 | 14 | 14 | 1231894 | 1196482 | 18781 | 16631 | 97.13 | 1.52 | 1.35 |
| 250 | 02/05 | 2 | 250_2_0205 | 16 | 16 | 1246508 | 1205690 | 24364 | 16454 | 96.73 | 1.95 | 1.32 |
| 250 | 02/06 | 2 | 250_2_0206 | 18 | 18 | 1249361 | 1205371 | 28498 | 15492 | 96.48 | 2.28 | 1.24 |
| 250 | 02/07 | 2 | 250_2_0207 | 18 | 18 | 1254429 | 1212302 | 26698 | 15429 | 96.64 | 2.13 | 1.23 |
| 250 | 02/08 | 2 | 250_2_0208 | 18 | 18 | 1246073 | 1209115 | 22877 | 14081 | 97.03 | 1.84 | 1.13 |
