# Supplementary material for: Three-dimensional organ segmentation and structural phenotyping of salt-stressed coriander seedlings using the optimized point transformer-based model PTV-SegCo
Source: Front Plant Sci. 2026 Aug 31;17:1883203. doi: 10.3389/fpls.2026.1883203 (PMC13572714; doi:10.3389/fpls.2026.1883203)
Supplement: Supplementary file 2 [file Table2.docx]

Table S2. Sensitivity analysis of the DBSCAN neighborhood radius for individual-plant separation

Detected cluster counts are reported for 20 representative scenes at five candidate neighborhood radii. MinPts was fixed at 20 and clusters containing fewer than 50 points were excluded. The final batch analysis used ε = 0.19 cm.

| **Scene** | **NaCl (mmol/L)** | **Date** | **Tray** | **Detected plant clusters** | | | | | **Manual interpretation** |
| --- | --- | --- | --- | --- | --- | --- | --- | --- | --- |
|  |  |  |  | **ε=0.16** | **ε=0.18** | **ε=0.19** | **ε=0.20** | **ε=0.22** |  |
| 0_2_0207 | 0 | 02/07 | 2 | 21 | 21 | **21** | 21 | 21 | Stable count |
| 0_2_0208 | 0 | 02/08 | 2 | 20 | 20 | **20** | 20 | 20 | Stable count |
| 50_1_0205 | 50 | 02/05 | 1 | 22 | 22 | **22** | 22 | 22 | Stable count |
| 50_1_0206 | 50 | 02/06 | 1 | 22 | 22 | **22** | 22 | 22 | Stable count |
| 50_1_0207 | 50 | 02/07 | 1 | 25 | 25 | **25** | 25 | 25 | Stable count |
| 50_1_0208 | 50 | 02/08 | 1 | 24 | 24 | **24** | 24 | 24 | Stable count |
| 50_2_0206 | 50 | 02/06 | 2 | 22 | 22 | **22** | 22 | 22 | Stable count |
| 50_2_0207 | 50 | 02/07 | 2 | 23 | 23 | **23** | 23 | 23 | Stable count |
| 50_2_0208 | 50 | 02/08 | 2 | 23 | 23 | **23** | 23 | 23 | Stable count |
| 100_1_0205 | 100 | 02/05 | 1 | 23 | 23 | **23** | 23 | 23 | Stable count |
| 100_1_0206 | 100 | 02/06 | 1 | 23 | 23 | **23** | 23 | 23 | Stable count |
| 100_1_0207 | 100 | 02/07 | 1 | 22 | 22 | **22** | 22 | 22 | Stable count |
| 100_1_0208 | 100 | 02/08 | 1 | 22 | 22 | **22** | 22 | 22 | Stable count |
| 100_2_0208 | 100 | 02/08 | 2 | 17 | 17 | **17** | 17 | 17 | Stable count |
| 150_2_0205 | 150 | 02/05 | 2 | 18 | 18 | **18** | 18 | 18 | Stable count |
| 200_2_0206 | 200 | 02/06 | 2 | 25 | 25 | **25** | 25 | 25 | Stable count |
| 200_2_0207 | 200 | 02/07 | 2 | 25 | 25 | **25** | 25 | 25 | Stable count |
| 250_1_0208 | 250 | 02/08 | 1 | 21 | 21 | **20** | 20 | 20 | 21 clusters at ε=0.16–0.18; 20 at ε≥0.19. Smaller ε over-segmented one plant fragment. |
| 0_1_0204 | 0 | 02/04 | 1 | 19 | 19 | **19** | 19 | 19 | Stable count |
| 0_1_0208 | 0 | 02/08 | 1 | 19 | 19 | **19** | 19 | 19 | Stable count |

Note: Cluster counts were unchanged across all five ε values in 19 of 20 scenes. For 250_1_0208, 21 clusters were obtained at ε=0.16–0.18 cm and 20 at ε=0.19–0.22 cm; visual inspection showed that the additional small-ε cluster was a disconnected fragment of the same plant. MinPts=20; clusters with <50 points were excluded.
