# Supplementary material for: Three-dimensional organ segmentation and structural phenotyping of salt-stressed coriander seedlings using the optimized point transformer-based model PTV-SegCo
Source: Front Plant Sci. 2026 Aug 31;17:1883203. doi: 10.3389/fpls.2026.1883203 (PMC13572714; doi:10.3389/fpls.2026.1883203)
Supplement: Supplementary file 3 [file Table3.docx]

Table S3. Manual quality-control corrections applied after DBSCAN-based individual-plant separation

Only manually confirmed corrections are listed. MERGE denotes disconnected clusters confirmed to belong to the same biological plant; DROP denotes a confirmed impurity/noise cluster removed before phenotypic calculation.

| **NaCl (mmol/L)** | **Date** | **Tray** | **Sample ID** | **QC action** | **Source plant IDs** | **Final retained ID** | **Source point counts** | **Output point count** | **QC interpretation** |
| --- | --- | --- | --- | --- | --- | --- | --- | --- | --- |
| 0 | 02/04 | 1 | 0_1_0204 | MERGE | 6, 8 | 6 | 302;1,015 | 1,317 | Same biological plant; disconnected fragments |
| 0 | 02/06 | 1 | 0_1_0206 | MERGE | 6, 18 | 6 | 317;1,015 | 1,332 | Same biological plant; disconnected fragments |
| 200 | 02/05 | 1 | 200_1_0205 | DROP | 12 | - | 57 | 0 | Impurity/noise fragment; non-plant |
| 250 | 02/04 | 1 | 250_1_0204 | MERGE | 0, 4 | 0 | 2,496; 85 | 2,581 | Same biological plant; disconnected fragments |
| 250 | 02/04 | 1 | 250_1_0204 | MERGE | 14, 12 | 14 | 50;1,603 | 1,653 | Same biological plant; disconnected fragments |
| 250 | 02/04 | 1 | 250_1_0204 | MERGE | 17, 16 | 17 | 99; 90 | 189 | Same biological plant; disconnected fragments |
| 250 | 02/07 | 1 | 250_1_0207 | MERGE | 6, 8 | 6 | 1,336;435 | 1,771 | Same biological plant; disconnected fragments |
| 250 | 02/08 | 1 | 250_1_0208 | MERGE | 3, 4 | 3 | 762;278 | 1,040 | Same biological plant; disconnected fragments |

Note: Seven merge operations and one impurity-cluster removal were applied after manual review of the pseudo-colored individual-plant point clouds in CloudCompare. These corrections reduced the total number of plant observations from 1,145 raw DBSCAN clusters to 1,137 QC-corrected observations. Phenotypic traits were recalculated from the corrected point sets.
